# Dietary Fiber-Induced Microbial Short Chain Fatty Acids Suppress ILC2-Dependent Airway Inflammation

**DOI:** 10.3389/fimmu.2019.02051

**Published:** 2019-09-18

**Authors:** Gavin Lewis, Bowen Wang, Pedram Shafiei Jahani, Benjamin P. Hurrell, Homayon Banie, German R. Aleman Muench, Hadi Maazi, Doumet Georges Helou, Emily Howard, Lauriane Galle-Treger, Richard Lo, Swetha Santosh, Andrew Baltus, Gerrold Bongers, Lani San-Mateo, Frank D. Gilliland, Virender K. Rehan, Pejman Soroosh, Omid Akbari

**Affiliations:** ^1^Janssen Research and Development, San Diego, CA, United States; ^2^Department of Molecular Microbiology and Immunology, Keck School of Medicine, University of Southern California, Los Angeles, CA, United States; ^3^Janssen Research and Development, Spring House, PA, United States; ^4^Division of Environmental Health, Department of Preventive Medicine, University of Southern California, Los Angeles, CA, United States; ^5^Division of Neonatology, Harbor-UCLA Medical Center, David Geffen School of Medicine at UCLA, Los Angeles, CA, United States

**Keywords:** dietary fiber, short chain fatty acid, ILC2, airway hyperreactivity, allergic disease

## Abstract

Group 2 Innate lymphoid cells (ILC2) contribute significantly to allergic inflammation. However, the role of microbiota on ILC2s remains to be unraveled. Here we show that short chain fatty acids (SCFAs), such as butyrate, derived from fermentation of dietary fibers by the gut microbiota inhibit pulmonary ILC2 functions and subsequent development of airway hyperreactivity (AHR). We further show that SCFAs modulate GATA3, oxidative phosphorylation, and glycolytic metabolic pathways in pulmonary ILC2s. The observed phenotype is associated with increased IL-17a secretion by lung ILC2s and linked to enhanced neutrophil recruitment to the airways. Finally, we show that butyrate-producing gut bacteria in germ-free mice effectively suppress ILC2-driven AHR. Collectively, our results demonstrate a previously unrecognized role for microbial-derived SCFAs on pulmonary ILC2s in the context of AHR. The data suggest strategies aimed at modulating metabolomics and microbiota in the gut, not only to treat, but to prevent lung inflammation and asthma.

## Introduction

Allergic disease affects 10–15% of people in the U.S. reflecting dysregulated immunity toward otherwise harmless environmental antigens. Recent studies have shown increased ILC2 activity in asthma and allergic diseases (1, 2). Group 2 innate lymphoid cells (ILC2s) produce type 2 cytokines such as IL-5, IL-13, and IL-9 in response to a growing number of environmental signals and epithelial cell-derived alarmins. In murine models of asthma, ILC2s are sufficient to provoke eosinophilic inflammation accompanied by airway hyperreactivity (AHR) independent of adaptive immunity (3). Finding negative regulators of ILC2 function remains an important clinical goal (1).

Both direct recognition of microbes as well as microbial metabolites have profound effects on immune system function including barrier defense, pathogen protection (4) and immune tolerance (5). Environmental stress including frequent use of antibiotics or high fat, low fiber diets are associated with increased incidence of many common autoimmune diseases including allergy and asthma alongside gut microbiome dysbiosis (6–8). For example, mice treated with antibiotics early in life or Germ-free mice were more prone to obesity and asthma, a finding also observed in human cohorts (9–11). Importantly, high-fiber diet or supplementation can restore both dysbiosis and protect against asthma in both human and animal models, the mechanism of which are incompletely understood (8, 12–14). Antibiotics (and Germ-free mice) also have substantial changes in ILC subset identity and epigenetic landscape in the small intestine, suggesting ILCs respond to microbial signals (15).

Short-chain fatty acids (SCFA) are a dependent product of dietary fiber fermentation by specific microbes in the intestinal colon and possesses multiple anti-inflammatory properties both on gut epithelial and immune cells (16, 17). SCFA bind G-protein coupled receptors GPR41 and 43 with varying affinity and can decrease pro-inflammatory cytokine expression by inhibiting the NFκB pathway and enhancing anti-microbial peptide secretion (16). Alternately GPCR signaling in non-hematopoietic cells can promote inflammatory response in the gut (18). In immune cells, butyrate inhibits Histone deacetylases (HDAC) to promotes Regulatory T cell (Treg) differentiation, and can induce IL-10 secretion in a GPR109 dependent manner in macrophages, protecting from colitis (19, 20). Systemic SCFAs can also decrease inflammation in the lungs, in part mediated by propionate- GPR43 receptor signaling on dendritic cells to express PDL-1 (13). The role of dietary fiber-induced butyrate and microbial metabolites on ILCs, in particular on ILC2s in the context of asthma, remains unknown.

Studies from our group and others show that ILC2s play a key role in the development of asthma pathogenesis. Following challenge with IL-25, IL-33, or allergens such as Alternaria and house dust mite (HDM), pulmonary ILC2s rapidly produce copious amounts of interleukin (IL)-5 and IL-13, which in turn leads to eosinophilia and mucous production and ultimately development of AHR (21). ILC2s may also promote tissue remodeling during chronic asthma in the lung via the secretion of amphiregulin (22). In addition to epithelial alarmins, lipid mediators produced by eosinophils and mast cells, such as prostaglandins PDGE and PGD2 can synergize with alarmins to activate cytokine receptor expression and ILC2 activity (23–25). Importantly ILC2s retain plasticity, as cytokines IL-12 and IL-1β convert ILC2 into ILC1 or Notch ligand DLL-1 converts ILC2 into ILC3-like cells (26–28).

Here we found that butyrate, derived from microbial fermentation of high fiber diet, significantly suppresses the production of type 2 cytokines by ILC2s *in vitro* and relieves ILC2-dependent allergic inflammation *in vivo*. Transcript analysis revealed GATA-3, a key transcription factor in ILC2 development and function, was significantly down-regulated after treatment with butyrate. Lowering GATA3 expression reduced cellular metabolism, limiting oxidative phosphorylation and glycolytic potential. We further determine that introducing endogenous butyrate production by specific microbiota abrogated ILC2-dependent AHR. Reduced type II response was also associated with increased IL-17a by ILC2 and enhanced neutrophil recruitment to the lungs. Overall, we found a mechanism of a well-studied bacterial metabolite important in gut function and extend its reach to regulation of cells in a distal organ such as the lung. This connection offers a unique way the environment can regulate immune reactivity, including innate lymphoid cells.

## Materials and Methods

### Mice

BALB/c and RAG^−/−^, and RAG^−/−^yC^−/−^ (all BALB/c background) mice were purchased from Taconic (Germantown, NY) and provided food and water *ad libitum* and be maintained on a 12 h light and dark cycle in the vivarium throughout study. All animal studies were approved by the Institutional Animal Care and Use Committee of Janssen R&D or USC and conducted in accordance with the USC Department of Animal Resources' guidelines. To induce allergic inflammation, mice received 0.5 ug recombinant mouse IL-33 (carrier-free, R&D) intranasally (*i.n.)* in 50 uL under isoflurane for 3 consecutive days. For diet studies, 5 week-old mice were place on normal chow (AIN-93, 4–5% cellulose Fiber content (w/w), or 30% cellulose or 30% Pectin, Research Diets, Inc.) for at least 2 months and weight monitored (Table S1). For mono-colonization studies, two high butyrate producer strains: *Clostridium butyricum* ATCC 19398C and *Clostridium sporogenes* ATCC 11437 and two low butyrate producing strains: *Clostridium ramosum* ATCC 13937 and *Clostridium ramosum* VPI 0427 ATCC 25582, were cultured anaerobically. Cultures were centrifuged, washed, and re-suspended in anaerobic solution (PBS) and frozen at −80°C until use. Germ-free mice received three gavages with 1 × 10^6^ PFU of high or low butyrate producing strains every other day in 200 uL PBS and subsequently exposed to 0.5 ug intranasal IL-33 for 3 consecutive days. Twenty four hours after the last exposure, lung function, bronchial alveolar lavage (BAL) and lung biopsies of recipients were analyzed.

### SCFA Measurement

Supernatant from Bead beater (Omni) homogenized Lung and Colon were Methanol extracted with 10 uM deuterated Free Fatty acid mix (Sigma) and derivatized with 3NPH (20 mM 3-nitrophenylhydrazine, 1-Ethyl-3-(3-dimethylaminopropyl) carbodiimide (EDC) 18 mM, Pyridine 1.5%, in 75% acetonitrile in water) for 30 min and 1 uL loaded for LCMS on C18 column in Agilent 1290 HPLC/6550 qTOF (Agilent, San Diego Ca) as previously described (29).

### Bacterial 16s

V4 rDNA sequencing and analysis was performed by Diversigen from frozen fecal pellets of *n* = 10 individual mice as previously described (30). Data are deposited as SRA.

### *Ex-vivo* Incubation of FACS-Purified ILC2s

ILC2 were purified from the lungs of IL-33 treated mice, 24 h after last challenge, as described previously (31, 32). Lungs were finely chopped and digested in 1 mg/mL Collagenase IV (MP Biomedicals, LLC) and DNaseI (Roche) for 30 min at 37°C followed by passage through 70 uM filter and pelleted with 30% Percoll to remove debris. ILC2 were FACS purified with ARIA Fusion cell sorter (BD Biosciences) gated as Live CD45^+^ Lineage^−^ Thy1-2^+^ CD127^+^ ST2^+^ to >95% purity. 5 × 10^3^ cells/well were cultured *ex-vivo* in RPMI (Lonza) with 10% FCS, HEPES, L-Glutamine, B-mercaptoethanol for 2–3 days in the presence of 10 ng/mL rmIL-2, rmIL-7, and 10 ng/mL IL-33 as indicated (R&D systems). Sodium chloride, acetate, propionate, and butyrate (Sigma) were dissolved in PBS pH 7.4 and added to the indicated concentrations. For knockdown studies, 1 uM GATA3 *in-vivo* morpholinos 5′-TGGTCCGCAGTCACCTCCATGTCCT-3′ or 5′mismatch control 5′-TGcTCCcCAcTCACCTCgATcTCCT-3′ (Gene Tools, Philomath OR) were added as free uptake oligos 24 h before butyrate addition. For GPR109a knockdown studies, translation blocking morpholino 5′- CTAGAAAATGGTCTGACTTGCTCAT-3′ and 5′mismatch control 5′- CTTGTAAATCGTCTCACTTCCTCAT-3′.

### Flow Cytometry

For BAL fluid collected, leukocyte populations were gated as previously described (33). Single cell suspensions of lung cells were stained for ILC2 as Live CD45^+^ Lineage^−^ Thy1-2^+^ CD127^+^ ST2^+^. For cytokine detection cells were stimulated for 4 h with PMA Ionomycin and Brefeldin A (cell stimulation cocktail, Biolegend) or BrefeldinA alone. After 15 min of viability stain with GhostRED (Tonbo, San Diego CA), cell fixation and intracellular cytokine staining was performed according to manufacturer instructions (BD biosciences San Jose CA) using IL-5-BV421, IL-13-APC, IL-17a-PeCy7 (Biologend, San Diego CA). For Transcription factor staining, FOXP3 fixation permeabilization kit was used according to manufacture and stained with GATA3-PECy7, ki67-e450, and IRF4-PE (eBioscience San Diego, CA). For mitochondrial analysis, ILC2 cultured with 20 nM MitoTrackerGreen and 100 nM MitoTrackercmxROS-H_2_ or 5 uM mitoSOX (Invitrogen, Carlsbad CA) in PBS with GhostRED dye for 30 min at 37°C 5% CO_2_. Cells were acquired on BD Canto II and analyzed with FlowJo software (Treestar Ashland OR).

### Cytokine Detection

BAL fluid, lung homogenate, or ILC2 culture supernatants were analyzed for presence of cytokines using mouse 32-plex Luminex kit (Millipore, Burlington MA), run on XMAP plate reader, and quantity of cytokine was calculated from standard curve reported as pg/mL using Masterplex software (Miraibio Inc) and graphed in Prism Software (GraphPad Software Inc).

### Seahorse

50,000–150,000 activated ILC2 were plated onto CellTak (BD Biosciences) coated microplates in Seahorse media supplemented with 1 mM Pyruvate, 2 mM glutamine, and 10 mM Glucose followed by mito stress test kit performed according to manufacture (Agilent, San Diego CA), using 1 uM FCCP.

### Adoptive Transfer

Experiments were performed as described previously (31). Briefly, activated ILC2 from IL-33 treated RAG^−/−^ mice were isolated as above and cultured for 48 h in the presence of 200 uM Sodium Chloride or Sodium Butyrate and 5 × 10^4^ cells transferred *i.v*. in PBS 1X into RAG^−/−^γC^−/−^ mice. Twenty four hours later, 0.5 μg rmIL-33 *i.n*. in 50 μL was given once a day for 3 days. AHR was then measured on day 4.

### Measure of Airway Hyperreactivity

Experiments were performed as described previously (32, 34). Lung function was evaluated by direct measurement of lung resistance and dynamic compliance in restrained tracheostomized mechanically ventilated mice using the FinePointe RC system (Buxco Research Systems, Wilmington, NC) under general anesthesia as described before (32). AHR was measured by exposure to an aerosol containing increasing doses of Methacholine (Sigma), following a baseline measurement after the delivery of a saline aerosol.

### Human ILCs

Purified from fresh Leukapheresis blood from healthy human donors, collected with IRB approval. Samples were depleted with Lineage depletion cocktail (Miltenyi) followed by cell sorting using FACS ARIA Fusion (BD biosciences, San Jose, CA) to purity >95%. Total ILCs were stained and gated as Live, CD45^+^ lineage^−^ (CD1a, CD3, CD14, CD16, CD19, CD20, CD56, CD123, and CD235a), CD127^+^, CD161^+^ and ILC2 sorted as: CRTH2^+^, CD117^+/−^ (all antibodies from eBioscience, San Diego CA). After isolation, 5 × 10^3^ ILCs were cultured in complete RPMI with 10% FCS and 10 ng/mL rhIL-2, 10 ng/mL rhIL7 and stimulated with 30 ng/mL rhIL-33 (R&D systems) for 5 days. Cell supernatants were analyzed for cytokine production with human 41-plex cytokine kit (Millipore) and read on XMAP Luminex plate reader, MFIs normalized to absolute values with provided standard curve using masterplex software and concentrations compared across individual donors by percent of untreated control. Cells were then fixed and stained intracellular GATA3 using FOXP3 perm/fixation kit (eBioscience) according to manufacture instructions and were acquired on BD Canto II and analyzed with FlowJo software (Treestar Ashland OR) for percentage and Median Florescence intensity compared in Prism software (GraphPad Software Inc., La Jolla, CA) using paired *T*-test.

### Statistical Analysis

Experiments were repeated at least 2–3 times (*n* = 4–8 per group) and data are shown as representative of independent experiments. A Bonferroni adjusted *t*-test for unpaired data was used for *in-vitro* samples. For *in-vivo* data, a Kruskal-Wallis test with Benjamini-Hochberg adjusted FDR or Dunn-corrected *P*-value was calculated using Prism Software (GraphPad Software Inc.). The degrees of significance were indicated as: ^*^p < 0.05, ^**^p < 0.01, ^***^p < 0.001.

## Results

### Dietary Fiber Dampens ILC2 Mediated Allergic Inflammation

**Figure 1:**
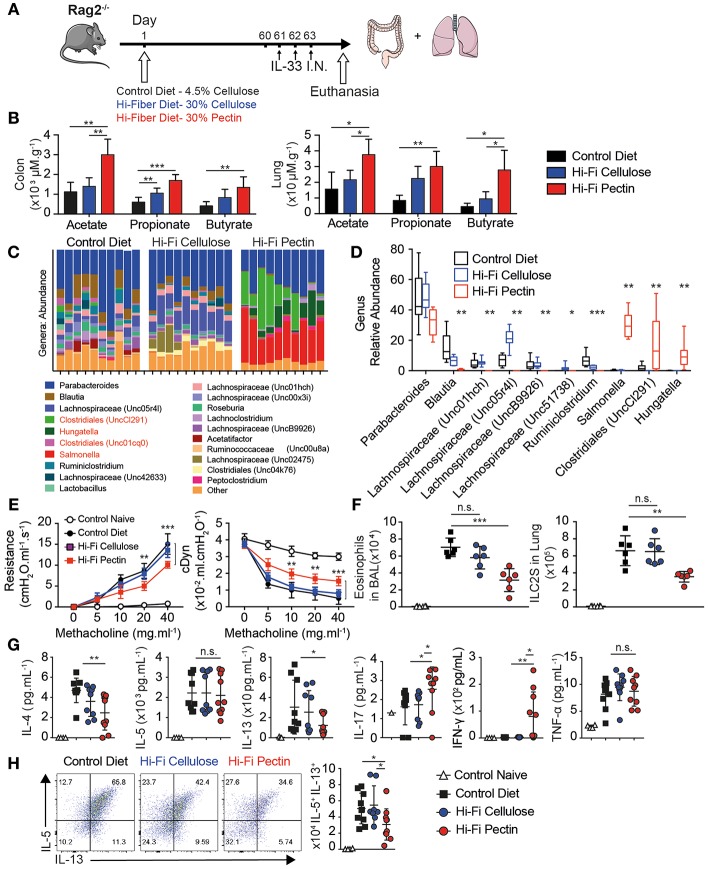
High-fiber diets dampens allergic asthma. Five week-old RAG^−/−^ were provided normal chow with 4.5% cellulose (Control Diet) or enriched with 30% cellulose (Hi-C) or 30% Pectin (Hi-P) as fiber source. After 2 months mice were treated with IL-33 intranasally (*i.n*.) for 3 consecutive days, followed by analysis of lung inflammation by flow cytometry. **(A)** Schematic. **(B)** Amounts of acetate, propionate, and butyrate in colon and lung tissue by LCMS. **(C)** Genus abundance by OTU count in fecal pellet by 16S V4 profiling in individual mice as columns. **(D)** Average proportions of most abundant bacterial genera. **(E)** Resistance (cmH_2_0/mL/S) and Dynamic compliance (mL/cmH_2_0) in anesthetized tracheotomized mice challenged with increasing dose methacholine. **(F)** Number of Eosinophils and ILC2 infiltrating BAL fluid. **(G)** BAL cytokine production by Luminex. **(H)** Intracellular cytokine production by Lung ILC2 after 12 h of IL-33 followed by Brefeldin A (BFA) of total lung cells, gated as Live CD45^+^Lineage^−^CD90.2^+^CD127^+^ST2^+^. Shown are representative plots and total number of IL-5 vs. IL-13 producing cells in lung. Data representative of two of four independent experiment, *n* = 4–5 mice/group+-SD. ^*^p < 0.05, ^**^p < 0.01, and ^***^p < 0.001.

Dietary Fiber derived short chain fatty acids modulate inflammation at distal sites including allergic airway inflammation in the lung. However, most models require suppressing T cell function through IL-10, PDL1, or FOXP3 regulation. To address if dietary fiber can modulate lung inflammation in absence of T cells, we assigned 5 week-old RAG2^−/−^ mice to a Normal 4.5% cellulose chow (Control Diet), or diets high in Cellulose (Hi-C) or Pectin (Hi-P) at 30% of dietary formula followed by allergic asthma challenge (Figure 1A). Pectin is more fermentable than cellulose and leads to increased SCFA production *in vivo*. The colon contained millimolar levels of acetate, propionate, and butyrate whereas lungs contained much lower micromolar levels of the three main SCFAs (35). Two months of Pectin diet significantly increased all SCFA levels, particularly butyrate (Figure 1B), without changes in normal weight over controls (Figure S1A).

Butyrate is preferentially derived by Phyla Firmicutes in mice, containing Clostridia clusters IV and XIVa. We next checked changes to microbiota in diet modified mice by 16S ribosomal RNA sequencing amplified from fecal pellets of individual mice. Phylogenetically, Pectin diet increased Proteobacteria such as Salmonella, as well as multiple different Firmicutes including genera Clostridiales, and Hungatella over Control or Cellulose diet, but decreased Lachnospriaceae by Operational Taxonomic Unit (OTU) count (Figure 1C). Interestingly, Pectin fed mice had decreased microbial diversity compared to control or Cellulose-fed mice by Shannon species diversity index (Figure S1B). Weighted Principal coordinate analysis (UniFrac distance) also showed Pectin-fed microbiome as distinct from both Cellulose and Control diet (Figure S1C). Indeed, there were significant changes in proportion of 9 out of 10 of the most abundant bacteria by OTU between controls and Pectin fed mice (Figure 1D). These results indicate Pectin diet allows outgrowth of a distinct microbiota associated with increased SCFA abundance.

Short term exposure to intranasal IL-33 induces ILC2 dependent eosinophilia and lung inflammation characteristic of allergic asthma in absence of T cells. After 3 daily doses, lungs of diet-modified mice were analyzed for Airway Hyperreactivity (AHR) by direct measurement of lung resistance and dynamic compliance in tracheostomized mechanically ventilated mice. Upon increasing methacholine challenge, Pectin fed mice had lower Resistance and increased Dynamic compliance than either control or cellulose-fed mice, indicating fiber diet moderately but significantly reduced the physiological features of allergic asthma (Figure 1E).

Next we analyzed Bronchial Alveolar Lavage (BAL) fluid for immune infiltration. There was a large influx in total number and percentage of Eosinophils in intranasal IL-33 in control mice and was decreased with Hi-Pectin diet compared to both normal chow and Hi-Cellulose control diets (Figure 1E). In turn, neutrophils were over-represented in percentage (Figure S1D) but not in overall number in mice fed hi-Pectin diets compared to controls. Next, we examined the levels of inflammatory cytokines in BAL fluid. IL-33 induces high amounts of type II cytokines IL-5 and IL-13, followed by IL-4 and TNF-α in control mice. Hi-Pectin diet reduced amounts of IL-13 and IL-4, but not IL-5 or TNF-α inflammatory cytokines (Figure 1G). Interestingly, we found increased in IL-17a found in BAL of Pectin fed mice. There was also an increase in IFN-γ across Pectin fed mice (Figure 1G). These data suggest Pectin fed mice had a mixed immune response with elements of Type I, II, and III responses, whereas control mice had Type II dominant response.

Innate lymphoid cells are the major producers of type II cytokines in this model (3). IL-33 induced ILC2 activation and expansion in the lungs of control diet fed mice, gated as CD45^+^ Lineage^−^ (CD3e, B220, Gr1, CD11b, Ter119, DX5, FcRe1) CD90.2^+^ CD127^+^ ST2^+^. Hi-Pectin diet had reduced numbers of ILC2 in the lungs compared to control diet fed mice (Figure 1F and Figure S1E). To further analyze ILC2 function, we cultured total lung cells overnight with IL-33, followed by 4 h stimulation with Brefeldin A to trap cytokine release, and measured intracellular cytokine production on ILC2 by flow cytometry. While the majority of activated ILC2 in control diet-fed mice produce IL-5 and IL-13, the total number of cytokine producing cells is decreased by hi-fiber diets, particularly IL-13 (Figure 1H). Next, we examined GATA3 expression in the lung ILC2 and found reduced GATA3 expression in Hi-Pectin diet groups compared to expression control group in the lungs where ILCs still strongly upregulated another transcription factor, IRF4 (Figure S1F). We did not see detectable changes in TBET or RORyT protein expression (Figure S1G). Overall these data indicate that increased levels of SCFA *in vivo* through diet can modulate the character of innate response to alarmin exposure in the lung, with reduced type II response and GATA3 expression.

### Butyrate Suppresses Activated ILC2 Function *in vitro* and *in-vivo*

**Figure 2:**
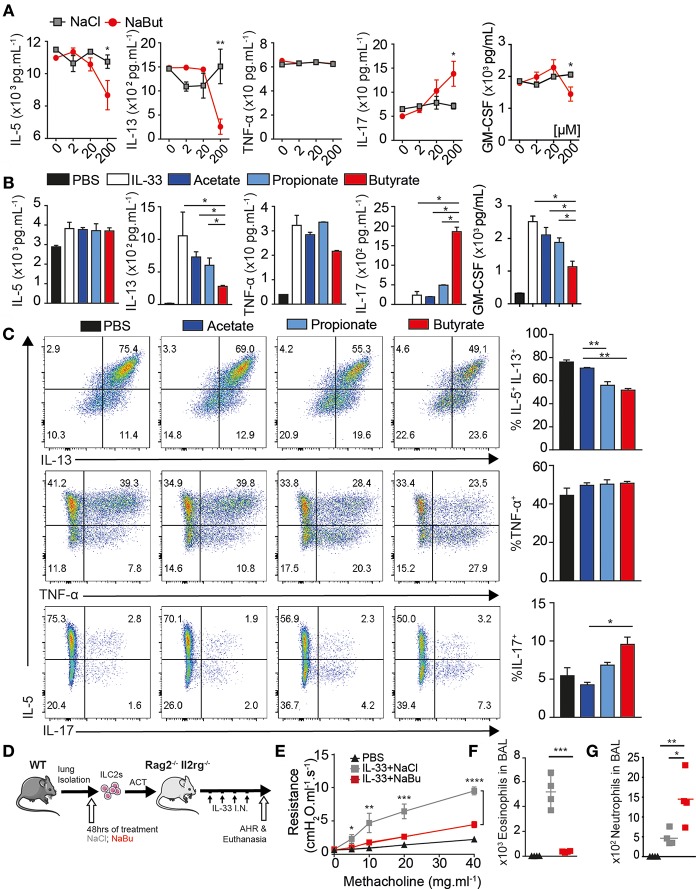
Butyrate suppresses ILC2 function *in-vitro* and *in-vivo*. ILC2s were FACS purified from lungs of mice with or without 3 days 0.5 ug IL-33 administration *i.n*. as indicated and 5 × 10^3^ cells/well-cultured *ex-vivo* for 2–3 days in the presence of IL-2 and IL-7, indicated salt at 2–200 uM and 10 ng/mL IL-33. **(A)** Schematic. **(B)** Dose dependence of ILC2 suppression by Sodium Butyrate compared to Sodium Chloride. **(C)** IL-5, IL-13, TNF-α, and IL-17a secretion in supernatant by activated ILC2 measured by Luminex after SCFA treatment at 200 uM. **(D)** Cytokine production by flow cytometry after 5 h stimulation with PMA/Ionomycin/BFA, comparing IL-5 and IL-13 (upper row), TNF-α (middle), or IL-17a in activated ILC2 after 3 days *in-vitro*. **(E)** Schematic, 5 × 10^4^ ILC2 treated with 200 uM NaCl or Butyrate, washed, transferred i.v. into RAG^−/−^γC^−/−^ mice challenged and with 3 days IL-33 *i.n*. **(F)** Lung resistance (AHR) upon increasing methacholine challenge. **(G)** Total Eosinophils in BAL. Total Neutrophil count in BAL. **(A–D)** Data are graphed as duplicate wells+-SEM, representative of three independent experiments and **(E–G)** two independent experiments, *n* = 4–5 mice/group+-SEM. ^*^p < 0.05, ^**^p < 0.01, ^***^p < 0.001, and ^****^p < 0.0001.

Given the observation of reduced ILC2 activity *in vivo* in response to IL-33, we next asked if activated ILC2 (aILC2) can respond directly to SCFA *ex vivo*. We cultured murine FACS-purified ILC2s as Live CD45^+^ Lineage^−^ CD90.2^+^ CD127^+^ CD25^+^ ST2^+^ (Figure S2A) from lungs of IL-33-treated mice *ex vivo* 48 h in the presence of IL-2, IL-7, and IL-33, and either sodium chloride or sodium butyrate at indicated physiologic concentration. Purified ILC2 secreted substantial amounts of IL-5 and IL-13 with IL-33 stimulation, measured in supernatants by Luminex. Butyrate treatment at 200 uM decreased IL-5 and IL-13 secretion in ILC2 when compared to the NaCl control group (Figure 2A). Sodium Butyrate did not affect viability, proliferation, or apoptosis below 1 mM (Figures S2B–D).

Butyrate also functions as a histone deacetylase inhibitor. We compared SCFA to HDAC inhibitor TSA and Quisinostat, a HDAC1 inhibitor. ILC2 underwent substantial apoptosis at 1 nM after 48 h whereas butyrate treatment did not induce similar effects (Figure S2E). When treated with Butyrate, activated ILC2 secreted less IL-13 and GM-CSF, but similar amounts of IL-5 and TNF-α into supernatant compared to acetate or propionate treated ILC2 (Figure 2B). Strikingly, purified ILC2 cultured with sodium butyrate secreted more IL-17a. We also tested cytokine production of aILC2 on a per cell basis by flow cytometry. Butyrate treatment, but not acetate or propionate, decreased intracellular both IL-5 and IL-13 production on a per cell basis. TNF-α production was not affected, consistent with Luminex data. Further, ILC2 that had lost IL-5 production start to produce IL-17a (Figure 2C). Activated ILC2 make copious amounts of IL-5 or may have stable IL-5 mRNA before Butyrate dampens secretion in supernatant, compared to PMA stimulation after 48 h where both IL-5 and IL-13 were reduced. ILC2 here were also capable of making small amount of IL-17a without butyrate, suggesting ILC2 have existing potential to make IL-17a, where IFN-y was not detected (not shown). These results demonstrate butyrate, among SCFA, is directly capable of modulating activated ILC2 function.

Increasing fiber in diet or Tributyrin supplementation can be beneficial for asthma patients, however Butyrate acts on many immune cells (12). Next, we tested whether butyrate treated ILC2s were sufficient to modulate disease *in vivo*. FACS-purified activated ILC2s (aILC2), characterized as above, were cultured 48 h *ex-vivo* with either sodium chloride or sodium butyrate. Washed cells were transferred i.v. into RAG^−/−^γC^−/−^ mice, followed by 3 daily doses of IL-33 *i.n*. to induce AHR (Figure 2D). Twenty four hours after last dose lung function was assessed by resistance and dynamic compliance. Recipient mice (RAG^−/−^γC^−/−^) lack all lymphocytes and do not otherwise develop AHR, where adoptive transfer of control treated ILC2 induces asthma-like symptoms upon methacholine challenge. However, butyrate treated ILC2 are unable to induce airway resistance upon transfer (Figure 2E). Following lung function measurement, BAL fluid was analyzed for eosinophil number by flow cytometry. Control-treated ILC2 strongly recruit eosinophils to the lungs upon IL-33 challenge. Consistent with AHR, butyrate-treated ILC2 did not recruit eosinophil to BAL (Figure 2F). In contrast, butyrate-treated ILC2 increased neutrophil recruitment to the lungs, consistent with IL-17a production (Figure 2G) (36). These results indicate that butyrate treatment on highly purified ILC2 is sufficient to limit pathogenic potential and modulate disease *in vivo*.

### Butyrate Regulates GATA3 and ILC2 Metabolism

**Figure 3:**
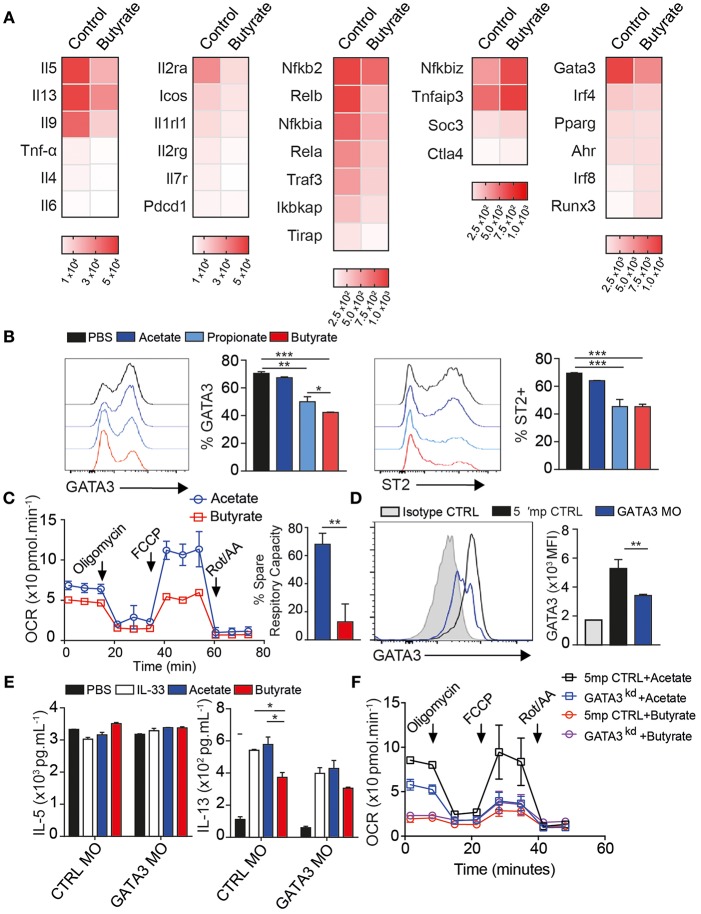
Butyrate regulates GATA3 expression and ILC2 metabolism. Activated ILC2s were FACS purified from lungs after 3 days IL-33 administration *i.n*. and cultured *ex-vivo* for 24 h in the presence of 200 uM Sodium Butyrate or Sodium Chloride as control for mRNA expression **(A)** or incubated with indicated SCFA sodium salt at 200 uM for 2–3 days **(B–D)**. **(A)** mRNA quantification of immune-related genes by Nanostring panel shown as heatmap of absolute expression counts. **(B)** Surface expression of ST2 and Intracellular expression of GATA3 in response to indicate SCFA. **(C)** Oxygen consumption measure (OCR) from Seahorse mito stress test 48 h post butyrate treatment. **(D–F)** Activated ILC2s were cultured *ex-vivo* for 2 days in the presence of 1 uM GATA3 Morpholino (GATA3 MO) or 5' mismatch control (CTRL MO) followed by another 2 days of Acetate or Butyrate treatment in presence of 10 ng/mL rmIL-33. **(D)** GATA3 expression over isotype. **(E)** IL-5 and IL-13 cytokine secretion in supernatant after acetate or butyrate addition. **(F)** Seahorse mito stress test assay after GATA3 knockdown ILC2 showing OCR and ECAR response. Data are graphed as duplicate-triplicate wells +-SEM, representative of 2–3 independent experiments. ^*^p < 0.05, ^**^p < 0.01, and ^***^p < 0.001.

To better understand the effects of butyrate, we next analyzed mRNA expression 24 h after *ex-vivo* stimulation, utilizing a panel of over 250 immune-related genes. ILC2 expressed prominent level of il5 and il13 mRNA. Butyrate treatment decreased il5, il13, and il9 expression by more than 50%, shown as heatmap of log2 absolute expression counts (Figure 3A, right). Whereas tnfa, il4, and il6 transcripts were expressed at much lower levels than il5 and il13, but also slightly reduced by butyrate treatment compared to control. Butyrate also decreased expression of il1r2 (ST2), il2ra (CD25), and icos but not pdcd1 (PD1) surface markers (middle panel) compared to control treated ILC2. In previous studies, sodium butyrate acted as an anti-inflammatory agent by inhibiting NFκB activation in human epithelial cells. Our transcript analysis showed butyrate also down-regulates nfκb1 and nfκb2 transcription as well as increased negative regulators tnfaip3 (A20) and socs3 (Figure 3A).

ILC2 require the transcription factor GATA3 and ST2 and IL-7r are GATA3 targets (37–39). Control ILC2 expressed high levels of gata3 and butyrate reduced gata3 within 24 h (Figure 3A-right). IRF4 is also important for IL-5 and IL-13 cytokine production and strongly induced in activated ILC2, however butyrate only slightly reduced irf4 transcripts. This suggests butyrate downregulates gata3 in ILC2. To confirm these results, we analyzed protein expression. The majority of untreated ILC2 express GATA3 in culture and expression was decreased with butyrate, and to lesser extent by propionate, but not acetate treatment (Figure 3B). There was no difference in viability or proliferation in these cultures (Figures S3A,B). Additional ILC2 surface ST2 and CD25, but not ICOS protein expression, were suppressed by Butyrate treatment, in line with mRNA results (Figure 3B and Figure S3C).

ILC2 utilize Long Chain Fatty acids over glucose for energy (40). To test if butyrate, as a carbon donor, altered cellular metabolism we first measured the amount of mitochondrial ROS generated using MitoSOX dye. Butyrate treated ILC2 generated less mitochondrial ROS than control treated cells (Figure S3D). We further analyzed the metabolic rate of ILC2 by Seahorse assay using mito stress test 48 h after butyrate addition. Resting metabolic rate of ILC2 was slightly reduced compared to acetate treated ILC2. However, butyrate completely ablated the spare respiratory capacity (SRC) of these cells after FCCP challenge, when compared to acetate treatment (Figure 3C). Interestingly, upon challenge with Oligomycin, ILC2 also had reduced ability to upregulate glycolytic activity (Figure S3E). We further tested propionate, which also did not reduce SRC compared to PBS treated ILC2 (Figure S3F). These data suggest Butyrate reduces the overall metabolic potential of ILC2 to utilize both OXPHOS and Glycolysis.

SCFAs have been reported to signal through GPCRs GPR41, 43, and 109a. We further measured mRNA expression by qPCR of gpr41, gpr43, and gpr109a. ILC2 express GPR109a over HPRT housekeeping gene but much lower levels of gpr41 and 43 (Figure S4A). We further measured Calcium flux by florescent indicator, Fura2, loaded into ILC2 followed by SCFA challenge. SCFA did not induce Ca^2+^ flux expressed by ILC2 regardless of GPR109a morpholino treatment, both groups responded to PMA control (Figure S4B). We then attempted knockdown of GPR109a, a single exon gene, using translation blocking morpholino, compared to 5' mismatch control. We measured IL-13 and IL-17a secretion by Luminex after treatment of cells with GPR109a Morpholino for 24 h followed by 48 h of butyrate or sodium chloride control. GPR109a morpholino treated ILC2 still produced less IL13 and more IL17a in response to butyrate, similar to Control morpholino (Figure S4C). We confirmed the function of ILC2 after GPR109a knockdown by 5 h of PMA stimulation in presence of BFA flowed by intracellular flow cytometry. GPR109a morpholino treated had similar IL-5 and IL-13-positive ILC2 as well as TNF-α-positive ILC2 as control morpholino treated after butyrate from 200 to 2 uM (Figure S4D). These data, alongside lack of effect of acetate and propionate as ligands suggest ILC2 do not utilize GPCR downstream of butyrate.

GATA3 is required for ILC2 to persist *in vivo* and produce IL-5 and IL-13 (39). To confirm acute downregulation of gata3 transcription was required for effects of butyrate we knocked down GATA3 protein (GATA3^KD^) by 48 h of splice-blocking morpholino. Targeted cells expressed 50% less GATA3 protein compared to 5' mismatch control morpholino treated cells, shown over isotype staining (gray), measured by intracellular flow cytometry (Figure 3D). GATA3^KD^ strongly reduced intracellular IL-5 and IL-13 on a per cell basis after PMA Ionomycin stimulation, graphed over Brefeldin A only control (gray), indicating GATA3 is required for continuous ILC2 function (Figure S5A). GATA3^KD^ cells had similar ki67 staining to control cells (Figure S5B). To examine if GATA3^KD^ also affected mitochondrial function, we again ran seahorse mito stress test on GATA3^KD^ ILC2. GATA3^KD^ ILC2 had decreased spare respiratory capacity after FCCP treatment, similar to butyrate treated ILC2 (Figure S5C).

To examine if butyrate could affect ILC2 in GATA3 sensitized background, we cultured GATA3^KD^ cells with SCFA for 3 days and measured cytokine production by Luminex. Again, IL-5 secretion was not affected by SCFA or GATA3^KD^, consistent with previous *in vitro* data (Figure 3E). However, IL-13 secretion was decreased by butyrate in control cells as expected and GATA3^KD^ reduced IL-13 secretion to levels similar to butyrate treatment (Figure 3E). Further, addition of butyrate to GATA3^KD^ had minimal further effect on IL-13, we measured membrane potential by flow cytometry using MitoTracker dye that binds actively respiring mitochondria at 48 h post stimulation. Addition of butyrate slightly decreased membrane potential (Figure S5D). Interestingly GATA3^KD^ also decreased mitochondrial membrane potential in IL-33 and acetate stimulated cells and addition of butyrate only had small effect on potential in GATA3 sensitized background. There was no change in mitochondrial size in staining with MitoTrackerGreen (Figure S5D). We next monitored real time mitochondrial function by Seahorse. Compared to control morpholino, GATA3^KD^ had slightly reduced basal respiration but again lacked SRC when challenged with Oligomycin and FCCP, similar to sodium butyrate treated ILC2 above in mito stress test (Figure 3F). Further treatment of GATA^KD^ with Butyrate reduced did not decrease SRC below GATA3^KD^. These data suggest indicating butyrate requires GATA3 to reduce ILC2 metabolic activity.

### Human ILC2 Are Suppressed by Butyrate

**Figure 4:**
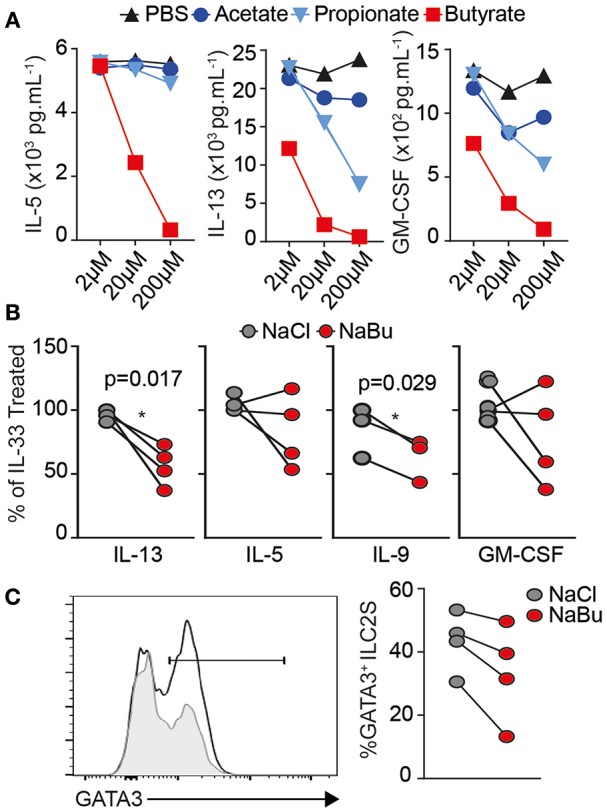
Human ILC2 are suppressed by Butyrate treatment. Human ILC2s were FACS purified (Live Lin^−^ CD45^+^ CD161^+^ CD127^+^ CRTh2^+^) from healthy human PBMCs and cultured *ex-vivo* for 5 days in the presence of 10 ng/mL rhIL-2, rhIL-7, and 30 ng/mL rhIL-33 with Sodium Chloride or Sodium Butyrate (10 uM unless indicated), at 5 × 10^3^ cells/well. **(A)** Dose titration and cytokine production in culture supernatant by Luminex assay, *n* = 2 donors. **(B,C)** Data are represented as percent of untreated controls, representative of four independent donors. **(C)** Intracellular GATA3 expression by flow cytometry in Acetate-treated (line) and butyrate (gray fill). Data representative of *n* = 6 independent donors ^*^p < 0.05 using paired *T*-test.

To extend our results to human we FACS-purified ILC2 from healthy donor PBMCs, as lineage^−^ CD45^+^ CD127^+^ CRTH2^+^ CD161^+^ (Figures S6A,B), cultured for 5 days in presence of recombinant human IL-2, IL-7, and IL-33 with indicated dose of SCFA. Similar to mouse, IL-33 activated human ILC2 produce substantial amounts of type 2 cytokines *in-vitro*. Butyrate, but not acetate or propionate, decreased type 2 cytokine secretion in a dose dependent manner (Figure 4A). We observed a significant decrease in IL-13 production and IL-9 across four individuals at 10 uM without affecting viability (Figure 4B). ILC2s from two patients showed a decrease in IL-5, the other two patients did not show a significance reduction in IL-5 after butyrate treatment, similar to mouse ILC2 where IL-5 was less affected than IL-13. We next tested whether human ILC2 also downregulated GATA3. Butyrate treatment also significantly decreased GATA3 protein expression compared to NaCl treatment, shown as representative overlay and percent positive paired across 4 donors (Figure 4C). These results suggest butyrate can also negatively regulate human ILC2 function via GATA3 expression.

### *Clostridia butyricum* Provides Systemic Butyrate to Modulate ILC2 Dependent AHR

**Figure 5:**
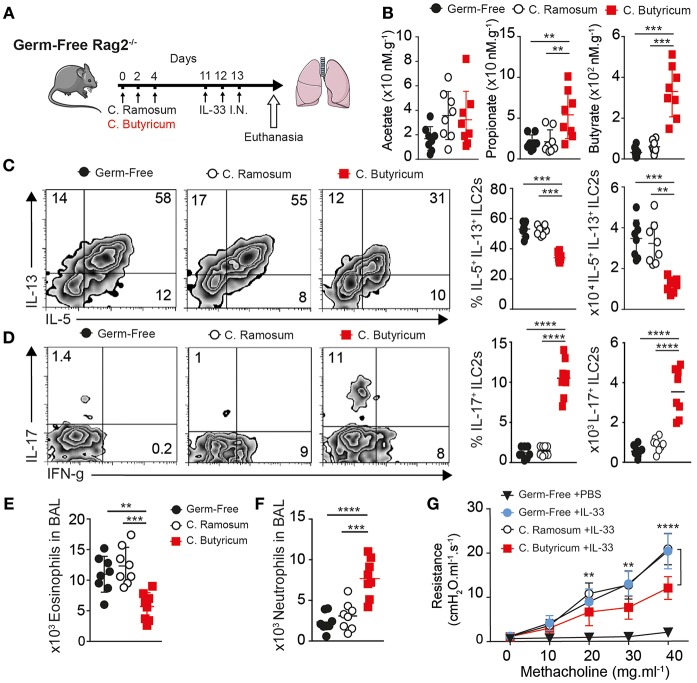
*Clostridia butyricum* provides systemic butyrate to dampen ILC2 dependent AHR. Germ-free RAG^−/−^ mice gavaged with 1 × 10^6^ pfu *Clostridia butyricum* and Clostridia *Sporogenes* (butyrate producer, BUT+) or two strains of *Clostridia ramosum* (non-butyrate producer, BUT–) every other day over 5 days, followed by once daily 0.5 ug rmIL-33 challenge intranasal for 3 days. Twenty four hours after last challenge, Lung function and composition was analyzed. **(A)** Schematic. **(B)** SCFA levels in Lung homogenate measured by LC-MS. **(C)** Representative flow plots, percentage and number of IL-5 and IL-13 producing ILC2 in BAL after PMA ionomycin/BFA. **(D)** Representative flow plots, percentage and number of IL-17a producing ILC2 as in **(C)**. **(E)** Total Eosinophils in BAL. **(F)** Total Neutrophil count in BAL. **(G)** Lung resistance (AHR) upon methacholine challenge. Data representative of two independent experiment, *n* = 8 mice/group+-SEM. ^*^p < 0.05, ^**^p < 0.01, ^***^p < 0.001, and ^****^p < 0.0001.

We found Fiber diet induced many changes in microbiota. We questioned if endogenous sodium butyrate produced by gut microflora was sufficient to abrogate AHR *in vivo* in an ILC2 dependent mouse asthma model. To this end, we took Germ-Free RAG^−/−^ mice (GF), with minimal microbiome exposure from birth, and gavaged 1 × 10^6^ PFU of fresh thawed aliquots of each of two butyrate producing strains of Clostridia species *(Clostridium butyricum* ATCC 19398C, *Clostridium sporogenes* ATCC 11437), labeled *C. butyricum* in legends, compared to gavage of two non-butyrate producing strains (*Clostridium ramosum* ATCC 13937, *Clostridium ramosum* VPI 0427 ATCC 25582) (41). We used two characterized strains each to increase engraftment and prevent artifacts of monocolonization. Following three gavages every other day, mice were exposed to intranasal IL-33 for 3 consecutive days and lungs analyzed 24 h after last challenge as diagramed (Figure 5A). To confirm effectiveness of gavage, we measured butyrate levels in whole lung homogenate (Figure 5B). Mice given butyrate producing Clostridia had increased levels of butyrate and propionate in lung, measured by LC-MS. Whereas, treatment with either Clostridia species increased acetate levels over germ-free controls.

Next, we looked at ILC2 number and function by flow cytometry in the lung. Interestingly butyrate producing Clostridia reduced both the percentage and total number of ILC2 producing IL-5 and IL-13 on per cell basis after PMA stimulation, when compared to *C. ramosum* control inoculated animals or GF controls where around 50% of ILC2 responded to produce IL-5 and IL-13 in assay (Figure 5C). To measure any shift in function we further look at IFN-γ and IL-17 production by ILC2. Consistent with previous data, ILC2 from lungs with increased butyrate had increased percentage and total number of IL-17a and IFN-γ producing cells after PMA Ionomycin stimulation (Figure 5D). Next, we analyzed the type of inflammatory cells infiltrating the lungs. Consistent with decreased IL-5, analysis of BAL fluid showed Clostridia producing butyrate reduced the number of eosinophils recruited to the lungs compared to control GF and *C. ramosum* gavaged mice (Figure 5E). In addition to decreased eosinophilia, *C. butyricum* gavage increased neutrophil numbers in the lungs compared to GF or control gavage (Figure 5F). Finally, *C. butyricum* gavage reduced physical airway resistance, upon measuring AHR with increasing methacholine challenge, compared to untreated GF mice or *C. ramosum* gavage controls (Figure 5G). Together these data suggest specific microbes that can produce butyrate in the gut influence ILC2 dependent lung inflammation and AHR *in vivo*.

## Discussion

Low fiber, high fat intake along with decreased microbial exposure are associated with increased development of autoimmune diseases and allergies such as asthma (42). Here we show that SCFAs derived from fermentation of dietary fibers by the gut microbiota, such as butyrate, can strikingly protect from ILC2-dependent Airway Hyperreactivity. SCFAs, as naturally occurring immune modulatory agents, are thought to have a wide variety of health benefits (43). Specifically, SCFAs in mice prevent obesity (44), reduce colitis by inducing colonic regulatory T cells (45), decrease pathology in models of asthma (13), and pathological bone loss (46). Most recently, in both mice and humans increasing Fiber consumption correlated decreased risk of Type II diabetes (47). Our results give mechanistic insight into the protective nature of dietary fiber-induced SCFAs on the development of asthma and allergic disease.

The colon and lungs of mice fed high Pectin diet were significantly enriched in acetate, propionate, and butyrate alongside large alterations in gut microbiota. The loss of diversity found here was also reported under Fiber diets in humans (47), where previously low microbial diversity is associated with dysbiosis. We found *in vivo* reconstitution of germ-free mice with specific butyrate-producing clostridia increased systemic butyrate exposure and reduced ILC2-dependent AHR in adult animals. This suggests short-term butyrate treatment might alleviate asthma symptoms. In confirmation, a recent publication claimed that administration of Sodium Butyrate in the drinking water may restrict pro-inflammatory cells via apoptosis in the context of airway inflammation, in a GPCR independent manner, similar to the HDAC inhibitor Tricostatin A (48, 49). However, butyrate is odorous and has low oral palatability and caused nasal inflammation in our hands in local delivery (not shown). Further, such histone deacetylase activity is genotoxic and may not be a physiological approach for long term treatment or prevention. We show that the production of butyrate by specific microbiota is a physiologic, non-invasive approach to treat allergic disease *in-vivo*.

Originally, isolated Clostridia species in mono-colonized mice and butyrate *in vitro* enhanced Treg differentiation through HDAC inhibitor function on FOXP3 locus. This protects against allergic airway disease and colitis in mice (20, 50). Acetate can similarly regulate HDAC activity in Tregs *in-vivo*, including *in utero* exposure through maternal diet (51). Increasing endogenous SCFA production by a high-fiber diet significantly also suppresses T cell immune responses in the lungs via propionate induced PDL1 on Bone Marrow derived Dendritic Cells in response to House Dust Mite (HDM) or Alum (13, 52). Interestingly our asthma modulation by butyrate occurs in RAG^−/−^ in absence of T cells, particularly Tregs. Clostridia also protect outgrowth of the fungal pathogen *Candida albicans* and subsequent macrophage derived prostaglandin PGD2 that can activate ILC2 (53). Whereas the presence of other bacteria, including *Lactobacillus johnsonii* and non-pathogenic strains of *Esherischia coli* are associated with increased type I immune responses (IFN-γ) and reduced Th2 response in lungs (54–56). Indeed, we found outgrowth of gram-negative Salmonella in Pectin treated mice and we detected increased IFN-γ in BAL of Pectin-fed mice and this might contribute to reduced ILC2 response (57).

Interestingly, these reports did not find increased neutrophils in BAL or lung alongside decreased eosinophilia and AHR. We found that high fiber diets led to a decrease of type-2 cytokine secretion by pulmonary ILC2s, and an increase in neutrophil recruitment in the lungs. Interestingly, a recent publication by Huang et al. reported inflammatory iILC2, induced by IL-25 or *N. brasiliensis* infection as KLRG^+^ ILC2, trafficked from lamina propria to the lungs during inflammation, rather than being local tissue derived nILC2 (58) where iILC2 were previously shown to have potential to make IL-17a (59). These data might support a direct link of microbiome metabolites exposure of ILC2 in the lamina propria, colon, or blood that then traffic to the lungs.

Shifts in immune response toward IL-17a driven neutrophilia have also been reported in chitin stimulated animals deficient in ILC2 derived cytokines IL-5 and IL-13 where ydT cells make IL-17a (60) and in Alternaria challenged IL-4r-deficient animals which lack GATA3 expression. We also found that purified ILC2s are capable of producing both IL-13 and IL-17a *in vitro*. Dual producing mouse ILC2 have been described after *in vitro* culture with notch ligands DLL1 as well as in human and non-human primate BAL samples (61, 62). Similarly, although obesity driven asthma shows an IL-17a signature alongside steroid resistance, neutrophils have been shown to be regulatory in asthma and hence butyrate-treated ILC2s might be anti-inflammatory overall by balancing the immune response away from pathogenic Th2 response (63). It remains open how the lung environment can contribute to ILC plasticity.

IL-33 is a potent activator of ILC2s, utilizing NFκB and MAPK signaling. We found reduced Rela and NFκB transcripts and increased tnfaip3 (A20) levels after butyrate treatment *in vitro*, consistent with reduced NFκB signaling. Transient modulation of GATA3 was also sufficient to reduce cytokine production by ILC2. Recently inducible deletion of GATA3, using ER-cre in adult mice, decreased ILC2 responses indicating a continuous requirement for GATA3 in a dose dependent manner (37, 39, 64). Inducible GATA3 deletion also showed GATA3 necessary for maintenance of IL-7r and c-myc expression in ILC2, which can drive metabolic growth pathways using glycolysis and oxidative phosphorylation. Indeed, IL-7r is important on memory CD4 T cells to maintain survival in part through metabolic programming (65). We found GATA3 was important for ILC2 to have both spare respiratory capacity and utilize glycolysis effectively. This might underscore the importance of GATA3 in all lymphocyte development and allow ILC2 to survive and respond effectively in periphery.

Butyrate undergoes beta-oxidation to increase acetate donor pool used for acetylation of both histone and non-histone proteins and where acetyl-CoA is not needed for TCA cycle, functions as HDAC inhibitor (66). Butyrate is also reported to be class III HDAC inhibitor of Sirtuins, where acetylation of PDHA1 suppresses complex I activity in mitochondria (67). Accumulation of acetylated H3 occurs in CNS1 region of FOXP3 locus, however the authors report no differences in acetylation status of gata3, tbx21, or rorc loci with butyrate in functionally similar CD4 T cells. We did not detect FOXP3 expression or changes in TBET or RORyT expression in ILC2. We found specific effects of butyrate on ILC2, and not acetate, arguing against HDAC activity and increased acetate levels as mechanism of action. Butyrate can also signal through a family of G-protein coupled receptors (GPCRs), GPR41, 43, and 109a in competition with acetate and propionate with different affinities, typically at millimolar concentrations. We also found acetate and propionate did not functionally influence ILC2 and SCFA did not induce Calcium flux inside cells, results that argue against receptor mediated GPCR activity. The direct link between Gata3 and metabolic function in ILC2 remains unknown.

Interestingly, ILC2s respond to many dietary metabolites and are important regulators of thermogenic brown adipose tissue in mice (68, 69). ILC2s rely on fatty acid oxidation (FAO) of long chain fatty acids for IL-13 production during *N. brasilienisis* infection (40). Similarly, a switch to glycolysis in VHL deficient mice, via HIF stabilization, can also suppress ILC2 function (70). Interestingly HIF stabilization or enhancing glycolysis can drive IL-17a production in CD4 T cells. We did not see increased glycolytic function after butyrate treatment and perhaps iILC2 prefer to increase arginase activity to provide polyamines for proliferation rather than glucose (71). Vitamin A is also important metabolite negatively regulating IL-13 function in ILC2, similarly through IL-7r response and decreasing spare respiratory capacity such that under starvation conditions of RA deficiency, IL-13 is increased to maintain barrier function (72). These results suggest butyrate, alongside Vitamin A and hypoxia as tissue specific signals that promote differentiation of ILC3 function in gut and conversely, accumulation of ILC2s in the lung environment. ILC2s, lacking other antigen specific receptors, might function primarily as metabolic sensors to skew immune responses (15, 73, 74).

The World Health Organization projects allergic disease associated with industrialization and western lifestyle to double over the next decade (75). Current therapeutic options for Th2-mediated diseases are limited to bronchodilators and immunosuppressive drugs that usually must be given indefinitely. Here we show dietary-derived butyrate inhibits ILC2s and subsequently reduces AHR, lung inflammation, and eosinophilia in a murine model of allergic asthma. Our data suggest that anti-inflammatory contributions of butyrate production and other microbial metabolites derived from our diet can be used not only to treat, but also to prevent lung inflammation and asthma.

## Data Availability

All datasets generated for this study are included in the manuscript and/or the Supplementary Files.

## Ethics Statement

All animal studies were approved by the Institutional Animal Care and Use Committee of Janssen R&D or USC and conducted in accordance with the USC Department of Animal Resources' guidelines.

## Author Contributions

PS and OA conceptualized and supervised the studies, contributed to data interpretation, and improvement of the manuscript. GL, BW, PS, BH, HB, GA, HM, SS, EH, LG-T, RL, AB, GB, and LS-M investigated. GL, PS, BH, and BW wrote original draft. OA, PS, GL, PS, FG, VR, and BH wrote, reviewed, and edited.

### Conflict of Interest Statement

GL, HB, GA, AB, GB, LS-M, and PS were employed by Janssen Pharmaceuticals. The remaining authors declare that the research was conducted in the absence of any commercial or financial relationships that could be construed as a potential conflict of interest.

## References

[1] Karta MR Broide DH Doherty TA. Insights into group 2 innate lymphoid cells in human airway disease. Curr Allergy Asthma Rep. (2016) 16:8. 10.1007/s11882-015-0581-6 26746844 PMC5026503

[2] Tait Wojno ED Artis D. Emerging concepts and future challenges in innate lymphoid cell biology. J Exp Med. (2016) 213:2229–48. 10.1084/jem.20160525 27811053 PMC5068238

[3] Kim HY Chang YJ Subramanian S Lee HH Albacker LA Matangkasombut P. Innate lymphoid cells responding to IL-33 mediate airway hyperreactivity independently of adaptive immunity. J Allergy Clin Immunol. (2012) 129:216–27 e211-16. 10.1016/j.jaci.2011.10.036 22119406 PMC3246069

[4] Pickard JM Zeng MY Caruso R Nunez G. Gut microbiota: role in pathogen colonization, immune responses, and inflammatory disease. Immunol Rev. (2017) 279:70–89. 10.1111/imr.12567 28856738 PMC5657496

[5] Kamada N Seo S-U Chen GY Núñez G. Role of the gut microbiota in immunity and inflammatory disease. Nat Rev Immunol. (2013) 13:321. 10.1038/nri3430 23618829

[6] Turnbaugh PJ Backhed F Fulton L Gordon JI. Diet-induced obesity is linked to marked but reversible alterations in the mouse distal gut microbiome. Cell Host Microbe. (2008) 3:213–23. 10.1016/j.chom.2008.02.015 18407065 PMC3687783

[7] Park Y Subar AF Hollenbeck A Schatzkin A. Dietary fiber intake and mortality in the NIH-AARP diet and health study. Arch Intern Med. (2011) 171:1061–8. 10.1001/archinternmed.2011.18 21321288 PMC3513325

[8] Wood LG Shivappa N Berthon BS Gibson PG Hebert JR. Dietary inflammatory index is related to asthma risk, lung function and systemic inflammation in asthma. Clin Exp Allergy. (2015) 45:177–83. 10.1111/cea.12323 24708388 PMC4190104

[9] Russell SL Gold MJ Hartmann M Willing BP Thorson L Wlodarska M. Early life antibiotic-driven changes in microbiota enhance susceptibility to allergic asthma. EMBO Rep. (2012) 13:440–7. 10.1038/embor.2012.32 22422004 PMC3343350

[10] Foliaki S Pearce N Björkstén B Mallol J Montefort S von Mutius E. Antibiotic use in infancy and symptoms of asthma, rhinoconjunctivitis, and eczema in children 6 and 7 years old: International Study of Asthma and Allergies in Childhood Phase III. J Allergy Clin Immunol. (2009) 124:982–9. 10.1016/j.jaci.2009.08.017 19895986

[11] Herbst T Sichelstiel A Schär C Yadava K Bürki K Cahenzli J. Dysregulation of allergic airway inflammation in the absence of microbial colonization. Am J Respir Crit Care Med. (2011) 184:198–205. 10.1164/rccm.201010-1574OC 21471101

[12] Halnes I Baines KJ Berthon BS MacDonald-Wicks LK Gibson PG Wood LG. Soluble fibre meal challenge reduces airway inflammation and expression of GPR43 and GPR41 in asthma. Nutrients. (2017) 9:57. 10.3390/nu9010057 28075383 PMC5295101

[13] Trompette A Gollwitzer ES Yadava K Sichelstiel AK Sprenger N Ngom-Bru C. Gut microbiota metabolism of dietary fiber influences allergic airway disease and hematopoiesis. Nat Med. (2014) 20:159–66. 10.1038/nm.3444 24390308

[14] Valcheva R Hotte N Gillevet P Sikaroodi M Thiessen A Madsen KL. Soluble dextrin fibers alter the intestinal microbiota and reduce proinflammatory cytokine secretion in male IL-10-deficient mice. J Nutr. (2015) 145:2060–6. 10.3945/jn.114.207738 26180249

[15] Gury-BenAri M Thaiss CA Serafini N Winter DR Giladi A Lara-Astiaso D. The spectrum and regulatory landscape of intestinal innate lymphoid cells are shaped by the microbiome. Cell. (2016) 166:1231–46 e1213. 10.1016/j.cell.2016.07.043 27545347

[16] McNabney SM Henagan TM. Short chain fatty acids in the colon and peripheral tissues: a focus on butyrate, colon cancer, obesity and insulin resistance. Nutrients. (2017) 9:E1348. 10.3390/nu9121348 29231905 PMC5748798

[17] Canani RB Costanzo MD Leone L Pedata M Meli R Calignano A. Potential beneficial effects of butyrate in intestinal and extraintestinal diseases. World J Gastroenterol. (2011) 17:1519–28. 10.3748/wjg.v17.i12.1519 21472114 PMC3070119

[18] Kim MH Kang SG Park JH Yanagisawa M Kim CH. Short-chain fatty acids activate GPR41 and GPR43 on intestinal epithelial cells to promote inflammatory responses in mice. Gastroenterology. (2013) 145:396–406.e310. 10.1053/j.gastro.2013.04.056 23665276

[19] Singh N Gurav A Sivaprakasam S Brady E Padia R Shi H. Activation of Gpr109a, receptor for niacin and the commensal metabolite butyrate, suppresses colonic inflammation and carcinogenesis. Immunity. (2014) 40:128–39. 10.1016/j.immuni.2013.12.007 24412617 PMC4305274

[20] Atarashi K Tanoue T Shima T Imaoka A Kuwahara T Momose Y. Induction of colonic regulatory T cells by indigenous *Clostridium* species. Science. (2011) 331:337. 10.1126/science.1198469 21205640 PMC3969237

[21] Walker JA McKenzie ANJ. Development and function of group 2 innate lymphoid cells. Curr Opin Immunol. (2013) 25:148–55. 10.1016/j.coi.2013.02.010 23562755 PMC3776222

[22] Monticelli LA Sonnenberg GF Abt MC Alenghat T Ziegler CG Doering TA. Innate lymphoid cells promote lung-tissue homeostasis after infection with influenza virus. Nat Immunol. (2011) 12:1045–54. 10.1038/ni.2131 21946417 PMC3320042

[23] Doherty TA Khorram N Lund S Mehta AK Croft M Broide DH. Lung type 2 innate lymphoid cells express cysteinyl leukotriene receptor 1, which regulates TH2 cytokine production. J Allergy Clin Immunol. (2013) 132:205–13. 10.1016/j.jaci.2013.03.048 23688412 PMC3704056

[24] Salimi M Stöger L Liu W Go S Pavord I Klenerman P. Cysteinyl leukotriene E4 activates human group 2 innate lymphoid cells and enhances the effect of prostaglandin D2 and epithelial cytokines. J Allergy Clin Immunol. (2017) 140:1090–100 e1011. 10.1016/j.jaci.2016.12.958 28115217 PMC5624780

[25] Xue L Salimi M Panse I Mjösberg JM McKenzie AN Spits H. Prostaglandin D2 activates group 2 innate lymphoid cells through chemoattractant receptor-homologous molecule expressed on TH2 cells. J Allergy Clin Immunol. (2014) 133:1184–94. 10.1016/j.jaci.2013.10.056 24388011 PMC3979107

[26] Lim AI Menegatti S Bustamante J Le Bourhis L Allez M Rogge L. IL-12 drives functional plasticity of human group 2 innate lymphoid cells. J Exp Med. (2016) 213:569–83. 10.1084/jem.20151750 26976630 PMC4821648

[27] Ohne Y Silver JS Thompson-Snipes L Collet MA Blanck JP Cantarel BL IL-1 is a critical regulator of group 2 innate lymphoid cell function and plasticity. Nat Immunol. (2016) 17:646–55. 10.1038/ni.3447 27111142

[28] Silver JS Kearley J Copenhaver AM Sanden C Mori M Yu L Inflammatory triggers associated with exacerbations of COPD orchestrate plasticity of group 2 innate lymphoid cells in the lungs. Nat Immunol. (2016) 17:626–35. 10.1038/ni.3443 27111143 PMC5345745

[29] Han J Lin K Sequeira C Borchers CH. An isotope-labeled chemical derivatization method for the quantitation of short-chain fatty acids in human feces by liquid chromatography-tandem mass spectrometry. Anal Chim Acta. (2015) 854:86–94. 10.1016/j.aca.2014.11.015 25479871

[30] Ajami NJ Cope JL Wong MC Petrosino JF Chesnel L. Impact of oral fidaxomicin administration on the intestinal microbiota and susceptibility to *Clostridium difficile* colonization in mice. Antimicrob Agents Chemother. (2018) 62:e02112–17. 10.1128/AAC.02112-17 29463537 PMC5923166

[31] Maazi H Banie H Aleman Muench GR Patel N Wang B Sankaranarayanan I. Activated plasmacytoid dendritic cells regulate type 2 innate lymphoid cell-mediated airway hyperreactivity. J Allergy Clin Immunol. (2018) 141:893–905.e896. 10.1016/j.jaci.2017.04.043 28579374

[32] Rigas D Lewis G Aron JL Wang B Banie H Sankaranarayanan I. Type 2 innate lymphoid cell suppression by regulatory T cells attenuates airway hyperreactivity and requires inducible T-cell costimulator-inducible T-cell costimulator ligand interaction. J Allergy Clin Immunol. (2017) 139:1468–77.e1462. 10.1016/j.jaci.2016.08.034 27717665 PMC5378695

[33] Maazi H Patel N Sankaranarayanan I Suzuki Y Rigas D Soroosh P. ICOS:ICOS-ligand interaction is required for type 2 innate lymphoid cell function, homeostasis, and induction of airway hyperreactivity. Immunity. (2015) 42:538–51. 10.1016/j.immuni.2015.02.007 25769613 PMC4366271

[34] Maazi H Singh AK Speak AO Lombardi V Lam J Khoo B. Lack of PD-L1 expression by iNKT cells improves the course of influenza A infection. PLoS ONE. (2013) 8:e59599. 10.1371/journal.pone.0059599 23555047 PMC3598698

[35] Holscher HD. Dietary fiber and prebiotics and the gastrointestinal microbiota. Gut Microbes. (2017) 8:172–84. 10.1080/19490976.2017.1290756 28165863 PMC5390821

[36] Ye P Rodriguez FH Kanaly S Stocking KL Schurr J Schwarzenberger P. Requirement of interleukin 17 receptor signaling for lung CXC chemokine and granulocyte colony-stimulating factor expression, neutrophil recruitment, and host defense. J Exp Med. (2001) 194:519–27. 10.1084/jem.194.4.519 11514607 PMC2193502

[37] Klein Wolterink RGJ Serafini N van Nimwegen M Vosshenrich CA de Bruijn MJ Fonseca Pereira D. Essential, dose-dependent role for the transcription factor Gata3 in the development of IL-5+ and IL-13+ type 2 innate lymphoid cells. Proc Natl Acad Sci USA. (2013) 110:10240–5. 10.1073/pnas.1217158110 23733962 PMC3690884

[38] Zhang D-H Cohn L Ray P Bottomly K Ray A. Transcription factor GATA-3 is differentially expressed in murine Th1 and Th2 cells and controls Th2-specific expression of the interleukin-5 Gene. J Biol Chem. (1997) 272:21597–603. 10.1074/jbc.272.34.21597 9261181

[39] Hoyler T Klose CS Souabni A Turqueti-Neves A Pfeifer D Rawlins EL. The transcription factor GATA-3 controls cell fate and maintenance of type 2 innate lymphoid cells. Immunity. (2012) 37:634–48. 10.1016/j.immuni.2012.06.020 23063333 PMC3662874

[40] Wilhelm C Harrison OJ Schmitt V Pelletier M Spencer SP Urban JF. Critical role of fatty acid metabolism in ILC2-mediated barrier protection during malnutrition and helminth infection. J Exp Med. (2016) 213:1409–18. 10.1084/jem.20151448 27432938 PMC4986525

[41] Narushima S Sugiura Y Oshima K Atarashi K Hattori M Suematsu M. Characterization of the 17 strains of regulatory T cell-inducing human-derived Clostridia. Gut Microbes. (2014) 5:333–9. 10.4161/gmic.28572 24642476 PMC4153770

[42] Ege MJ Mayer M Normand AC Genuneit J Cookson WO Braun-Fahrländer C. Exposure to environmental microorganisms and childhood asthma. N Eng J Med. (2011) 364:701–9. 10.1056/NEJMoa1007302 21345099

[43] Hand TW Vujkovic-Cvijin I Ridaura VK Belkaid Y. Linking the microbiota, chronic disease, and the immune system. Trends Endocr Metabol. (2016) 27:831–43. 10.1016/j.tem.2016.08.003 27623245 PMC5116263

[44] Lu Y Fan C Li P Lu Y Chang X Qi K. Short chain fatty acids prevent high-fat-diet-induced obesity in mice by regulating G protein-coupled receptors and gut microbiota. Sci Rep. (2016) 6:37589. 10.1038/srep37589 27892486 PMC5124860

[45] Smith PM Howitt MR Panikov N Michaud M Gallini CA Bohlooly-Y M. The microbial metabolites, short-chain fatty acids, regulate colonic Treg cell homeostasis. Science. (2013) 341:569–73. 10.1126/science.1241165 23828891 PMC3807819

[46] Lucas S Omata Y Hofmann J Böttcher M Iljazovic A Sarter K. Short-chain fatty acids regulate systemic bone mass and protect from pathological bone loss. Nat Commun. (2018) 9:55. 10.1038/s41467-017-02490-4 29302038 PMC5754356

[47] Zhao L Zhang F Ding X Wu G Lam YY Wang X. Gut bacteria selectively promoted by dietary fibers alleviate type 2 diabetes. Science. (2018) 359:1151. 10.1126/science.aao5774 29590046

[48] Toki S Goleniewska K Reiss S Zhou W Newcomb DC Bloodworth MH. The histone deacetylase inhibitor trichostatin A suppresses murine innate allergic inflammation by blocking group 2 innate lymphoid cell. (ILC2) activation. Thorax. (2016) 71:633–45. 10.1136/thoraxjnl-2015-207728 27071418 PMC4941189

[49] Thio CL Chi PY Lai AC Chang YJ. Regulation of type 2 innate lymphoid cell-dependent airway hyperreactivity by butyrate. J Allergy Clin Immunol. (2018) 142:1867–83.e12. 10.1016/j.jaci.2018.02.032 29522844

[50] Atarashi K Tanoue T Oshima K Suda W Nagano Y Nishikawa H. Treg induction by a rationally selected mixture of *Clostridia* strains from the human microbiota. Nature. (2013) 500:232. 10.1038/nature12331 23842501

[51] Thorburn AN McKenzie CI Shen S Stanley D Macia L Mason LJ. Evidence that asthma is a developmental origin disease influenced by maternal diet and bacterial metabolites. Nat Commun. (2015) 6:7320. 10.1038/ncomms8320 26102221

[52] Zhang Z Shi L Pang W Liu W Li J Wang H. Dietary fiber intake regulates intestinal microflora and inhibits ovalbumin-induced allergic airway inflammation in a mouse model. PLoS ONE. (2016) 11:e0147778. 10.1371/journal.pone.0147778 26872019 PMC4752249

[53] Kim YG Udayanga KG Totsuka N Weinberg JB Núñez G Shibuya A. Gut dysbiosis promotes M2 macrophage polarization and allergic airway inflammation via fungi-induced PGE. Cell Host Microbe. (2014) 15:95–102. 10.1016/j.chom.2013.12.010 24439901 PMC3957200

[54] Fonseca W Lucey K Jang S Fujimura KE Rasky A Ting HA. Lactobacillus johnsonii supplementation attenuates respiratory viral infection via metabolic reprogramming and immune cell modulation. Mucosal Immunol. (2017) 10:1569–80. 10.1038/mi.2017.13 28295020 PMC5599307

[55] Fujimura KE Demoor T Rauch M Faruqi AA Jang S Johnson CC. House dust exposure mediates gut microbiome *Lactobacillus* enrichment and airway immune defense against allergens and virus infection. Proc Natl Acad Sci USA. (2014) 111:805–10. 10.1073/pnas.1310750111 24344318 PMC3896155

[56] Pang W Wang H Shi L Sun Y Wang X Wang M. Immunomodulatory effects of *Escherichia coli* ATCC 25922 on allergic airway inflammation in a mouse model. PLoS ONE. (2013) 8:e59174. 10.1371/journal.pone.0059174 23536867 PMC3607577

[57] Wu CJ Chen LC Kuo ML. Attenuated Salmonella typhimurium reduces ovalbumin-induced airway inflammation and T-helper type 2 responses in mice. Clin Exp Immunol. (2006) 145:116–22. 10.1111/j.1365-2249.2006.03099.x 16792681 PMC1942008

[58] Huang Y Mao K Chen X Sun MA Kawabe T Li W. S1P-dependent interorgan trafficking of group 2 innate lymphoid cells supports host defense. Science. (2018) 359:114–19. 10.1126/science.aam5809 29302015 PMC6956613

[59] Huang Y Guo L Qiu J Chen X Hu-Li J Siebenlist U. IL-25-responsive, lineage-negative KLRG1(hi) cells are multipotential ‘inflammatory’ type 2 innate lymphoid cells. Nat Immunol. (2015) 16:161–9. 10.1038/ni.3078 25531830 PMC4297567

[60] Van Dyken SJ Mohapatra A Nussbaum JC Molofsky AB Thornton EE Ziegler SF. Chitin activates parallel immune modules that direct distinct inflammatory responses via innate lymphoid type 2 and gammadelta T cells. Immunity. (2014) 40:414–24. 10.1016/j.immuni.2014.02.003 24631157 PMC4019510

[61] Zhang K Xu X Pasha MA Siebel CW Costello A Haczku A. Cutting edge: notch signaling promotes the plasticity of group-2 innate lymphoid cells. J Immunol. (2017) 198:1798–803. 10.4049/jimmunol.1601421 28115527 PMC5321819

[62] Flayer CH Ge MQ Tompkins DG Juarez M Miller L Royer CM Group 2 innate lymphoid cells display ILC3-like functional plasticity in asthmatics and non-human primates. J Allergy Clin Immunol. (2018) 141:AB1 10.1016/j.jaci.2017.12.004

[63] Schnyder-Candrian S Togbe D Couillin I Mercier I Brombacher F Quesniaux V. Interleukin-17 is a negative regulator of established allergic asthma. J Exp Med. (2006) 203:2715–25. 10.1084/jem.20061401 17101734 PMC2118159

[64] Yagi R Zhong C Northrup DL Yu F Bouladoux N Spencer S. The transcription factor GATA3 is critical for the development of all IL-7Ralpha-expressing innate lymphoid cells. Immunity. (2014) 40:378–88. 10.1016/j.immuni.2014.01.012 24631153 PMC4026797

[65] Wofford JA Wieman HL Jacobs SR Zhao Y Rathmell JC. IL-7 promotes Glut1 trafficking and glucose uptake via STAT5-mediated activation of Akt to support T-cell survival. Blood. (2008) 111:2101–11. 10.1182/blood-2007-06-096297 18042802 PMC2234050

[66] Donohoe DR Collins LB Wali A Bigler R Sun W Bultman SJ. The Warburg effect dictates the mechanism of butyrate-mediated histone acetylation and cell proliferation. Mol Cell. (2012) 48:612–26. 10.1016/j.molcel.2012.08.033 23063526 PMC3513569

[67] Xu S Liu CX Xu W Huang L Zhao JY Zhao SM. Butyrate induces apoptosis by activating PDC and inhibiting complex I through SIRT3 inactivation. Signal Transduct Targeted Ther. (2017) 2:16035. 10.1038/sigtrans.2016.35 29263907 PMC5661613

[68] Lee MW Odegaard JI Mukundan L Qiu Y Molofsky AB Nussbaum JC. Activated type 2 innate lymphoid cells regulate beige fat biogenesis. Cell. (2015) 160:74–87. 10.1016/j.cell.2014.12.011 25543153 PMC4297518

[69] Moro K Yamada T Tanabe M Takeuchi T Ikawa T Kawamoto H. Innate production of T(H)2 cytokines by adipose tissue-associated c-Kit(+)Sca-1(+) lymphoid cells. Nature. (2010) 463:540–4. 10.1038/nature08636 20023630

[70] Li Q Li D Zhang X Wan Q Zhang W Zheng M. E3 Ligase VHL Promotes group 2 innate lymphoid cell maturation and function via glycolysis inhibition and induction of interleukin-33 receptor. Immunity. (2018) 48:258–70 e255. 10.1016/j.immuni.2017.12.013 29452935 PMC5828523

[71] Monticelli LA Buck MD Flamar AL Saenz SA Tait Wojno ED Yudanin NA. Arginase 1 is an innate lymphoid-cell-intrinsic metabolic checkpoint controlling type 2 inflammation. Nat Immunol. (2016) 17:656–65. 10.1038/ni.3421 27043409 PMC4873382

[72] Spencer SP Wilhelm C Yang Q Hall JA Bouladoux N Boyd A. Adaptation of innate lymphoid cells to a micronutrient deficiency promotes type 2 barrier immunity. Science. (2014) 343:432–7. 10.1126/science.1247606 24458645 PMC4313730

[73] Odegaard JI Chawla A. The immune system as a sensor of the metabolic state. Immunity. (2013) 38:644–54. 10.1016/j.immuni.2013.04.001 23601683 PMC3663597

[74] Wilhelm C Kharabi Masouleh S Kazakov A. Metabolic regulation of innate lymphoid cell-mediated tissue protection-linking the nutritional state to barrier immunity. Front Immunol. (2017) 8:1742. 10.3389/fimmu.2017.01742 29375541 PMC5770634

[75] Nunes C Pereira AM Morais-Almeida M. Asthma costs and social impact. Asthma Res Pract. (2017) 3:1. 10.1186/s40733-016-0029-3 28078100 PMC5219738

