# Supplementary material for: Dietary Fiber-Induced Microbial Short Chain Fatty Acids Suppress ILC2-Dependent Airway Inflammation
Source: Front Immunol. 2019 Sep 18;10:2051. doi: 10.3389/fimmu.2019.02051 (PMC6760365; doi:10.3389/fimmu.2019.02051)
Supplement: Supplementary file 1 [file Data_Sheet_1.PDF]

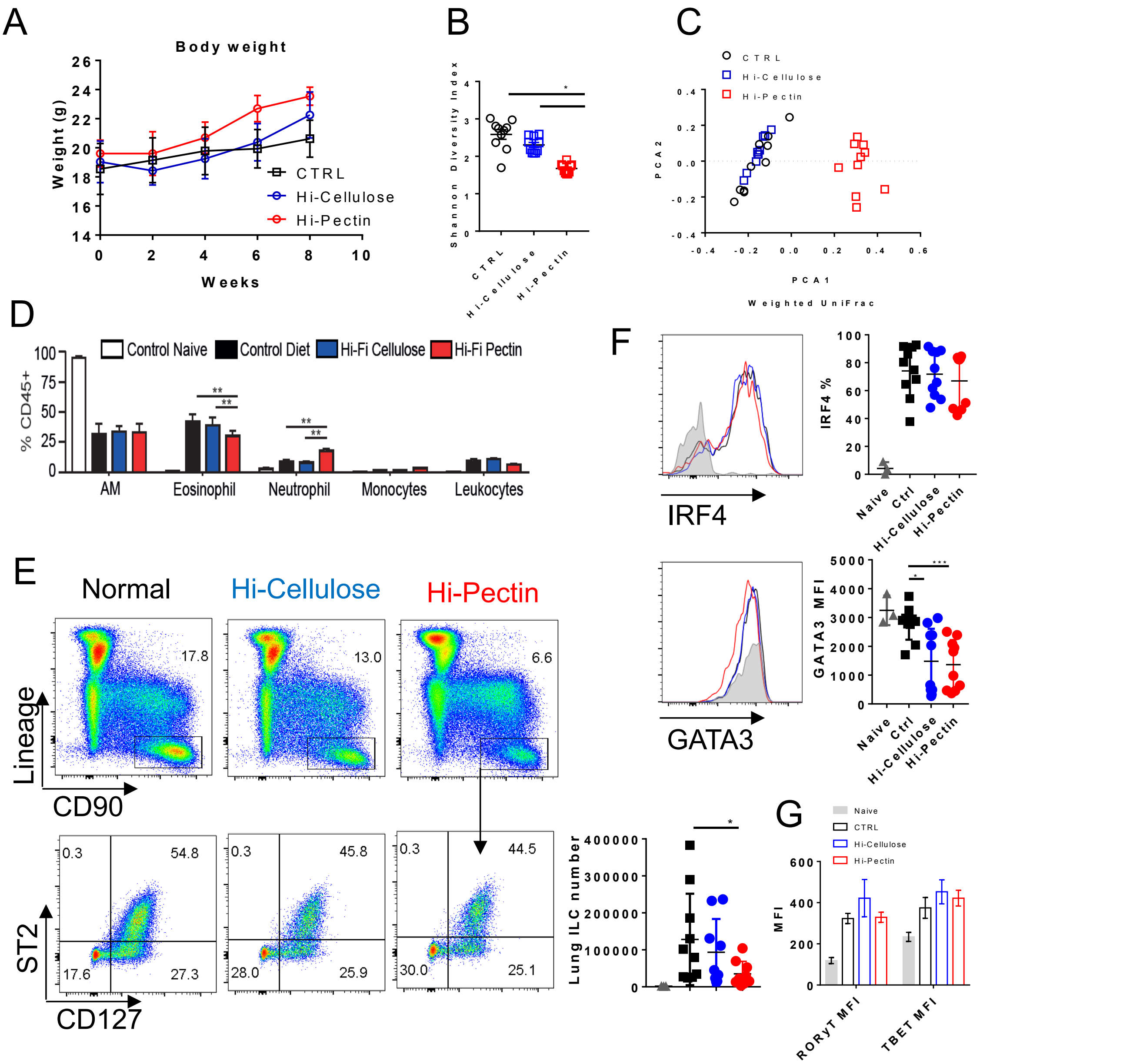

Fig S1

High-Fiber diets dampens allergic asthma

5-week-old $RAG^{-/-}$ were provided normal chow with 4.5% cellulose or enriched with 30% cellulose or 30% Pectin (w/w) as fiber source. After two months mice were treated with IL33 intranasal (i.n.) for three consecutive days, followed by analysis of lung inflammation by flow cytometry.

A) Weight over two months
B) 16S V4 rDNA profiling of individual fecal pellets. Shannon diversity index is shown.
C) Number of leukocytes infiltrating BAL fluid.
D) Total number of Eosinophils and Neutrophils in BAL
E) Flow cytometry of ILC2 in the lung, gated as Live $CD45^{+}Lineage^{-}CD90.2^{+}CD127^{+}ST2^{+}$. Shown are representative plots percentage and total number in lung.
F) IRF4 and GATA3 expression in ILC2 by intracellular flow cytometry, representative plots and MFI.
G) RORyT and TBET expression in ILC2 by intracellular flow cytometry.

Data representative of 2 independent experiment, n=5 mice/group * $p<0.05$, ** $p<0.005$.

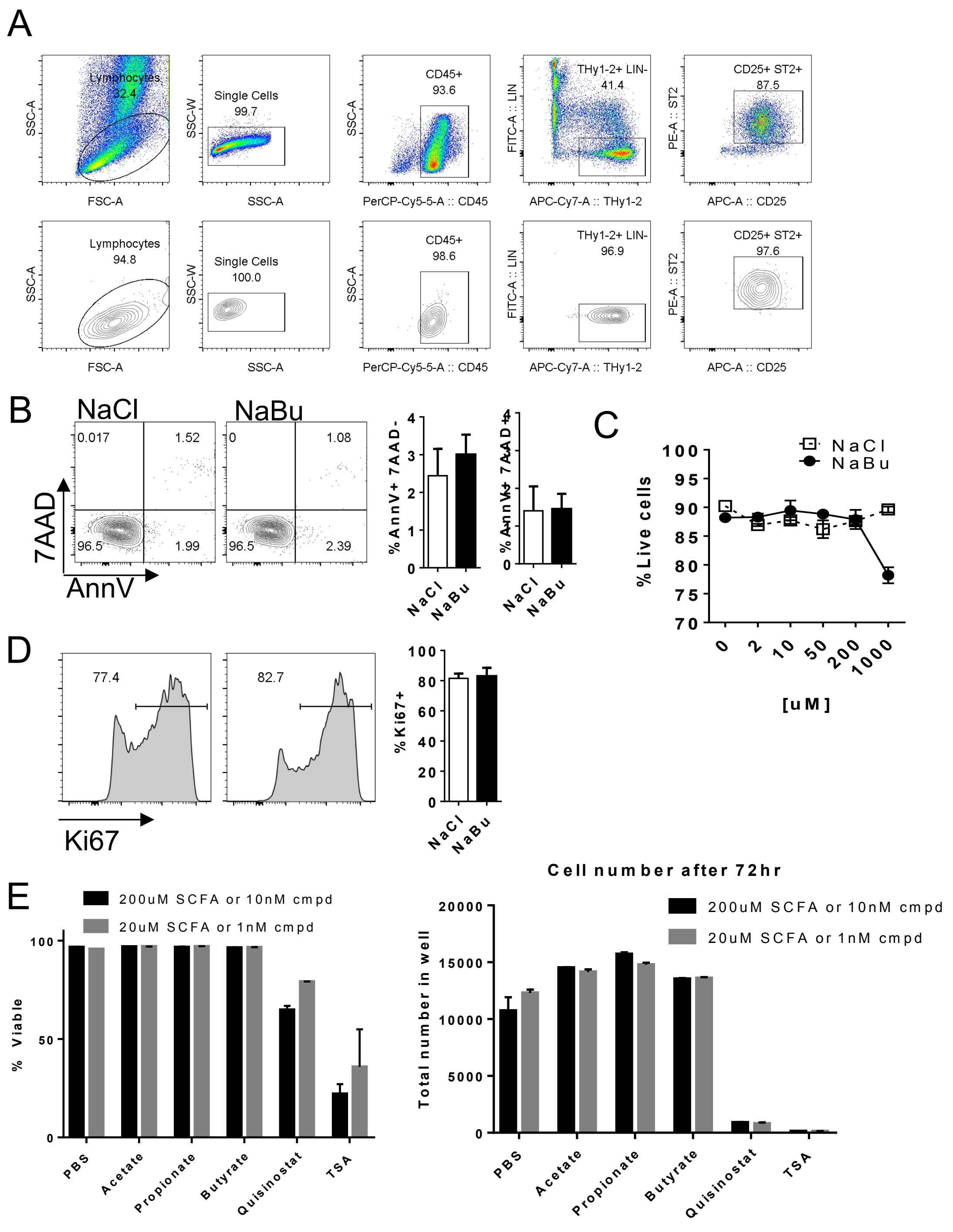

Figure S2. Butyrate suppresses ILC2 function in-vitro

A) ILC2s were FACS purified from lungs of mice with 3 days 0.5ug IL33 administration i.n. to >95% purity.

B) $5x10^3$ cells/well cultured ex-vivo for 2-3 days in the presence of IL2, IL7 and IL33, and indicated salt at 2-1000uM followed by AnnexinV and 7AAD staining

C) Percent viable over dose in (b)

D) Ki67 by intracellular flow cytometry

E) Percent viable and total number of ILC2 treated with 10nM or 1nM Trichostatin A or Quisinostat as indicated or 200uM or 20uM indicated SCFA for 48hrs and analyzed by flow cytometry.

Data are graphed as duplicate wells, representative of 3 independent experiments. * p<0.05, ** p<0.005.

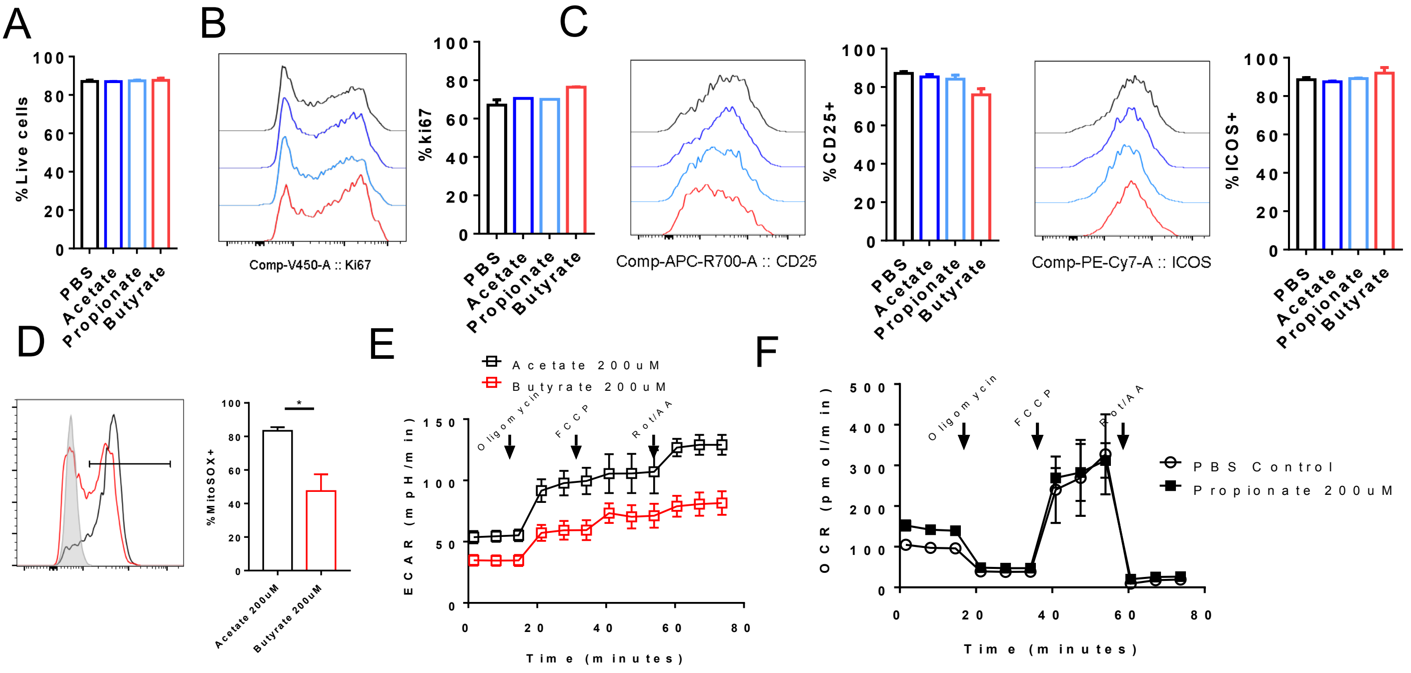

Figure S3. Butyrate regulates GATA3 expression in ILC2

Activated ILC2s were FACS purified from lungs after 3 days IL33 administration i.n. and cultured ex-vivo with indicated SCFA sodium salt at 200uM for 3 days. cultured ex-vivo for 2 days in the presence of 1uM GATA3 Morpholino (GATA3 MO) or 5' mismatch control (CTRL MO) followed by another 48hrs of Acetate or Butyrate treatment in presence of 10ng/mL rmIL33. Mitochondrial phenotype was assessed by flow cytometry.

a) Percentage Viable cells
b) Ki67 expression
c) CD25 and ICOS expression
d) MitoSOX staining after 30minutes at 37F
e) ECAR from Seahorse mito stress test 48hr post butyrate or acetate treatment
f) OCR and ECAR from Seahorse mito stress test 48hr post propionate treatment.

Data are graphed as duplicate-triplicate wells, representative of 2-3 independent experiments. * p<0.05, ** p<0.005.

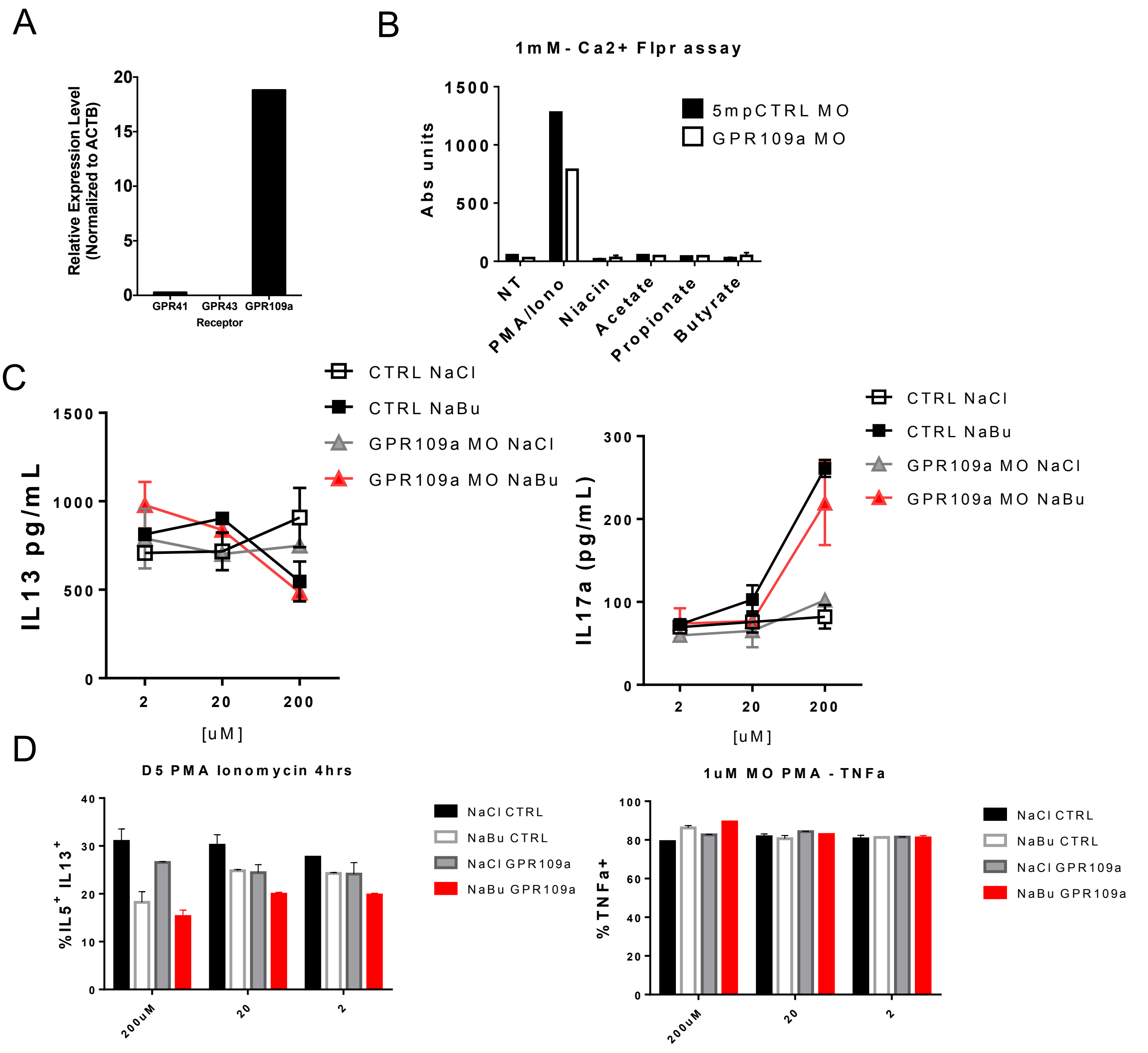

Figure S4. Butyrate does not require GPR109a to suppress ILC2

Activated ILC2s were FACS purified from lungs after 3 days IL33 administration i.n. and cultured ex-vivo for 1-2 days in the presence of 1uM GPR109a Morpholino (GPR109a MO) or 5' mismatch control (CTRL) followed by another 48hrs of Sodium Chloride (NaCl) or Sodium Butyrate (NaBu) treatment in presence of 10ng/mL rmIL33. Mitochondrial phenotype was assessed by flow cytometry.

A) GPR41,43,109a by qPCR in ILC2
B) Calcium flux measured by Furo-2 reporter after 1mM SCFA or 50ng/ml PMA and Ionomycin
C) Cytokine IL-13 and IL17a secretion by Luminex after indicated dose of SCFA.
D) Percentage of cells producing both IL5 and IL13 or TNFa after 5 hour stimulation with PMA/ Ionomycin in the presence of BFA.

Data are graphed as duplicate wells, representative of 2 independent experiments. * $p<0.05$, ** $p<0.005$.

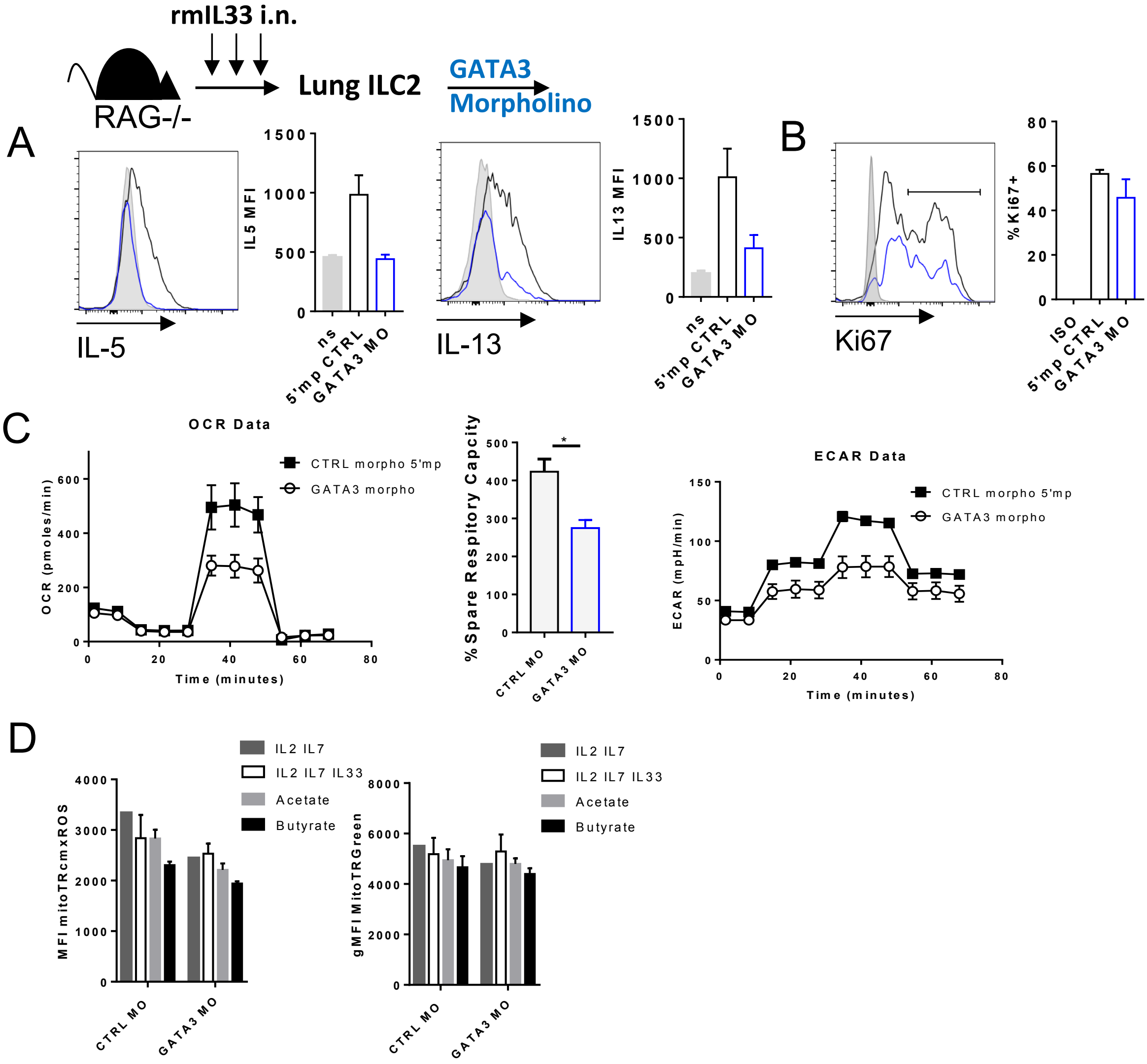

Figure S5. GATA3 regulates ILC2 metabolism.

Activated ILC2s were FACS purified from lungs after 3 days IL33 administration i.n. and cultured ex-vivo for 1-2 days in the presence of 1uM GATA3 Morpholino. Mitochondrial phenotype was assessed by flow cytometry.

A) IL5 and IL13 after 3hr PMA Ionomycin over no stimulation control (BFA only-grey)
B) Ki67 expression over isotype (grey)
C) OCR and ECAR from Seahorse mito stress test on $1x10^5$ ILC2 48hrs after GATA3 knockdown.
D) Mitochondrial size and membrane potential staining with 20nM MitoTrackerGreen or 100nM MitoTrackercmxROSH_2 by flow cytometry, graphed as MFI.

Data are graphed as duplicate wells, representative of 2 independent experiments. * $p<0.05$, ** $p<0.005$.

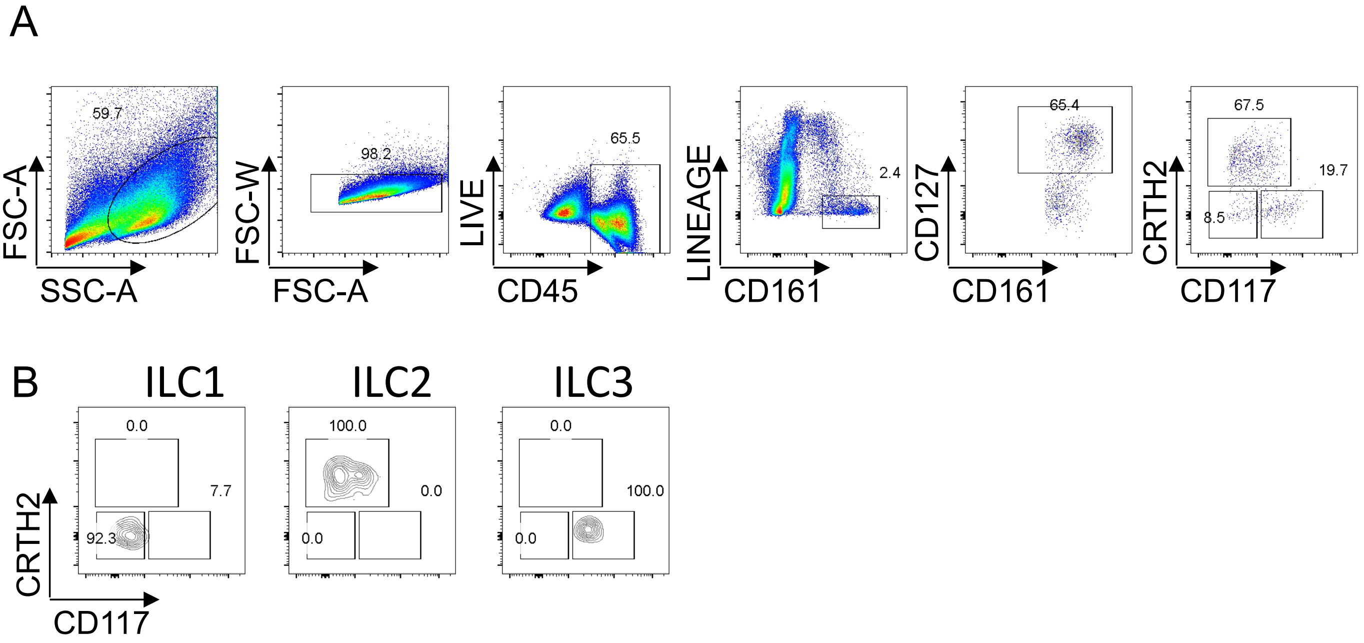

Figure S6. Butyrate suppresses human ILC2

Human ILCs were purified from fresh Luekophoresed blood with Lineage depletion cocktail followed by FACS to purity >95%.

A) Total ILC2s were sorted as Live, CD45^{+} lineage^{-} (CD1a, CD3, CD14, CD16, CD19, CD20, CD56, CD123, and CD235a), CD127^{+}, CD161^{+} CRTH2^{+}, CD117$^{+/-}$

B) After isolation, 5x10^{3} ILCs were cultured in complete RPMI with 10%FCS and 10ng/mL rhIL-2, 10ng/mL rhIL7 and stimulated with 30ng/mL rhIL33 (R&D systems) for 5 days.

Data representative of 6 independent donors.

Supplemental table 1. High fiber diet formulation provided by Research Diets, Inc

OpenSource DIETS®

Formulated by:
Research Diets, Inc.

D10012M and D16042501-04

AIN-93M Mature Rodent Diet and Same with Different Levels of Dietary Fiber (Cellulose or Pectin)

**Product #**	**D10012M**		**D16042501**		**D16042502**		**D16042503**		**D16042504**	
	AIN-93M		*Standard*		*Low Fiber*		*High Fiber with Cellulose*		*High Fiber with Pectin*	
	gm%	*kcal%*	gm%	*kcal%*	gm%	*kcal%*	gm%	*kcal%*	gm%	*kcal%*
Protein	14	*15*	14	*15*	15	*15*	10	*15*	10	*15*
Carbohydrate	73	*76*	73	*76*	77	*76*	54	*76*	54	*76*
Fat	4	*9*	4	*9*	4	*9*	3	*9*	3	*9*
Total		*100*		*100*		*100*		*100*		*100*
kcal/gm	3.8		3.9		4.0		2.8		2.8	
**Ingredient**	**gm**	***kcal***	**gm**	***kcal***	**gm**	***kcal***	**gm**	***kcal***	**gm**	***kcal***
Casein	140	*560*	140	*560*	140	*560*	140	*560*	140	*560*
L-Cystine	1.8	*7*	1.8	*7*	1.8	*7*	1.8	*7*	1.8	*7*
Corn Starch	495.692	*1983*	495.692	*1983*	495.692	*1983*	495.692	*1983*	495.692	*1983*
Maltodextrin 10	125	*500*	125	*500*	125	*500*	125	*500*	125	*500*
Sucrose	100	*400*	100	*400*	100	*400*	100	*400*	100	*400*
**Cellulose, BW200**	50	*0*	**44.77**	*0*	**2.89**	*0*	**407.15**	*0*	0	*0*
**Pectin, Citrus**	0	*0*	0	*0*	0		0	*0*	**407.15**	*0*
Soybean Oil	40	*360*	40	*360*	40	*360*	40	*360*	40	*360*
t-Butylhydroquinone	0.008	*0*	0.008	*0*	0.008	*0*	0.008	*0*	0.008	*0*
Mineral Mix S10022M	35	*0*	35	*0*	35	*0*	35	*0*	35	*0*
Vitamin Mix V10037	10	*40*	10	*40*	10	*40*	10	*40*	10	*40*
Choline Bitartrate	2.5	*0*	2.5	*0*	2.5	*0*	2.5	*0*	2.5	*0*
FD&C Yellow Dye #5	0	*0*	0	*0*	0.05	*0*	0	*0*	0	*0*
FD&C Red Dye #40	0	*0*	0	*0*	0	*0*	0.05	*0*	0	*0*
FD&C Blue Dye #1	0	*0*	0	*0*	0	*0*	0	*0*	0.05	*0*
**Total**	**1000**	***3850***	**994.77**	***3850***	**952.94**	***3850***	**1357.2**	***3850***	**1357.2**	***3850***
**Cellulose (%)**	**5.00**		**4.50**		**0.30**		**30.00**		**0.00**	
**Pectin (%)**	**0.00**		**0.00**		**0.00**		**0.00**		**30.00**	
